# The Mechanism for Processing Random-Dot Motion at Various Speeds in Early Visual Cortices

**DOI:** 10.1371/journal.pone.0093115

**Published:** 2014-03-28

**Authors:** Xu An, Hongliang Gong, Niall McLoughlin, Yupeng Yang, Wei Wang

**Affiliations:** 1 CAS Key Laboratory of Brain Function and Diseases, School of Life Sciences, University of Science and Technology of China, Hefei, P. R. China; 2 Institute of Neuroscience and State Key Laboratory of Neuroscience, Shanghai Institutes for Biological Sciences, Chinese Academy of Sciences, Shanghai, P. R. China; 3 Faculty of Life Sciences, University of Manchester, Manchester, United Kingdom; University College London, United Kingdom

## Abstract

All moving objects generate sequential retinotopic activations representing a series of discrete locations in space and time (motion trajectory). How direction-selective neurons in mammalian early visual cortices process motion trajectory remains to be clarified. Using single-cell recording and optical imaging of intrinsic signals along with mathematical simulation, we studied response properties of cat visual areas 17 and 18 to random dots moving at various speeds. We found that, the motion trajectory at low speed was encoded primarily as a direction signal by groups of neurons preferring that motion direction. Above certain transition speeds, the motion trajectory is perceived as a spatial orientation representing the motion axis of the moving dots. In both areas studied, above these speeds, other groups of direction-selective neurons with perpendicular direction preferences were activated to encode the motion trajectory as motion-axis information. This applied to both simple and complex neurons. The average transition speed for switching between encoding motion direction and axis was about 31°/s in area 18 and 15°/s in area 17. A spatio-temporal energy model predicted the transition speeds accurately in both areas, but not the direction-selective indexes to random-dot stimuli in area 18. In addition, above transition speeds, the change of direction preferences of population responses recorded by optical imaging can be revealed using vector maximum but not vector summation method. Together, this combined processing of motion direction and axis by neurons with orthogonal direction preferences associated with speed may serve as a common principle of early visual motion processing.

## Introduction

**Figure 1:**
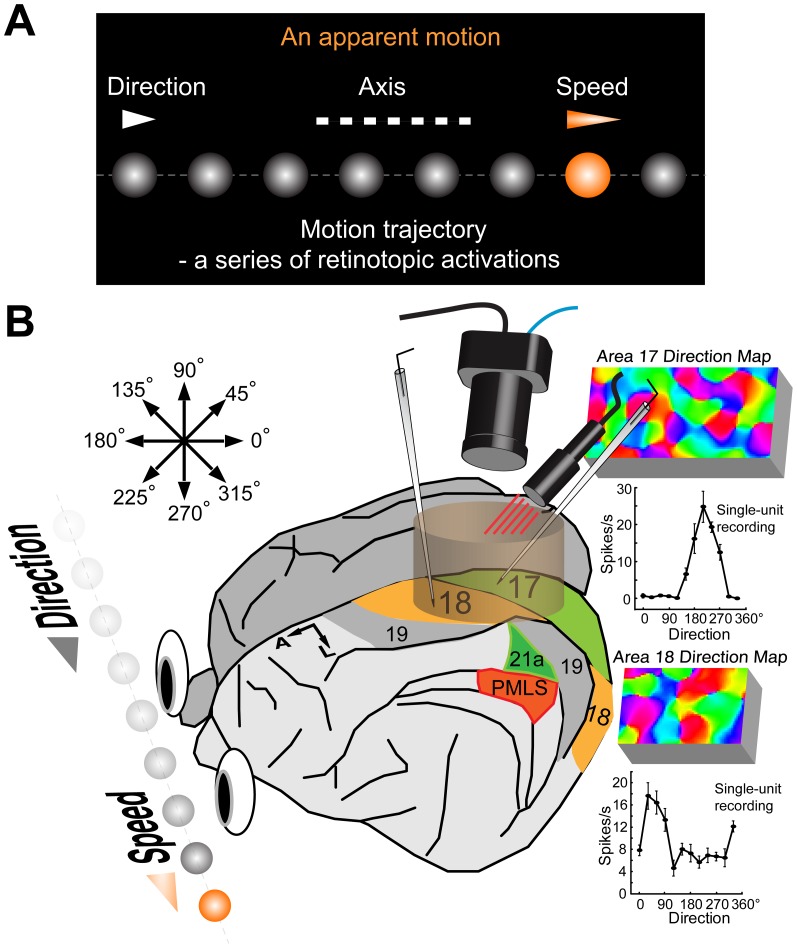
Schematic illustration of the research focus and the logic of the experiments undertaken. (A) A schematic diagram that illustrates motion direction, speed, and axis in the case of an apparent motion stimulus, in which each bulb is lighted sequentially in rightward direction at different times without any actual position changes (no real motion involved but a series of retinotopic activations in space and time, i.e. motion trajectory). The sequential retinotopic activations by flashes can lead to the perception of direction, axis, and speed. (B) The experimental approach. Direction-selective population and single-cell responses to random-dot motion from cat early visual areas 17 and 18 were acquired by the combination of intrinsic-signal optical imaging and single-unit recordings. Arrows inside the figure demonstrate the definition of motion direction that ranges from 0° to 360°. Note that motion axis is the same for dots moving in opposite directions and ranges from 0° to 180°. Examples of results from areas 17 and 18 are demonstrated.

Motion direction, speed, and axis are common features of any physical movement. These features can be accurately detected and perceived by human and non-human primate [1]–[2]. In addition, humans can also perceive apparent motion in the cinema as if it were generated by real moving objects (Figure 1A). This is because the motion stimulus elicits a series of retinotopic activations that represent a sequence of discrete locations in space and time (motion trajectory). These sequential activations enable the perception of motion direction [3]–[6]. When objects move above a certain speed, the individual locations of moving objects can no longer be resolved neurophysiologically. The motion trajectory therefore is perceived as a spatial orientation representing the motion axis (Figure 1A). This is commonly known as motion streak [7]–[8].

Human psychophysical studies have found that the motion streak signal generated by a moving white spot can aid direction discrimination when the speed exceeds a critical value [7], [9]. Using electrophysiological recordings in cat area 17, neuronal populations were first demonstrated to encode the trajectory of a fast moving dot as a spatial orientation signal at a speed of 38.4°/s [10]. A later study in primary visual cortex of cat and monkey found that a spot moving parallel to the preferred orientation of the cell was more effective for activating the cell at high, but not low speed [11]. Through probing population responses in ferret V1 with different parameters of moving random-bar stimuli, an optical imaging study showed that the preference of population responses of orientation-selective cells was speed dependent [12]. The results of this population study were later successfully simulated by spatio-temporal energy based models [13]–[14], suggesting that the responses of orientation-selective cells to moving stimuli in V1 can be understood from the linear properties of these cells. However, these early studies mainly focused on orientation-selective mechanism in motion processing but did not explicitly examine the contribution of direction-selective mechanisms especially under different speed conditions. As both orientation- and direction-selective cells are prevalent in early visual cortices, it is important to know the behavior of direction-selective cells in motion processing at high speed. Interestingly, only a subgroup of direction-selective cells in the primary visual cortex were found to exhibit “parallel motion direction selectivity”, supporting the idea that motion streak signals are present in V1 [11]. In a recent study [15], we demonstrated that at the population level orientation-selective neurons in V1, V2, and V4 of macaque ventral visual pathway can encode motion-axis information at high speed, thus contributing directly to the perception of motion streak. However, we observed, surprisingly, that the preference of direction-responsive domains in the thick stripes of V2 was independent of motion speed when calculated using vector summation. Thus, it also remains elusive as to how individual direction-selective neurons in different early visual cortices encode the motion trajectory at different speeds. Furthermore, as most studies only focused on the response property of the primary visual cortex, the response differences to motion axis between different visual areas still need to be addressed. Here, the above questions were specifically studied in cat areas 17 and 18, where direction-selective neurons are prevalent and cluster into iso-direction preference domains [16]–[20].

Using intrinsic signal optical imaging and single-unit recording, the neuronal responses to random-dot stimuli moving at various speeds were studied (Figure 1B). We found that for random dots moving in a given direction, direction-selective neurons preferring that direction were activated primarily at low speeds. By contrast, other groups of neurons preferring perpendicular directions were activated to signal exclusively the motion-axis information at high speeds. The speed, at which neurons with perpendicular direction preferences were activated for encoding motion-axis information, was defined as the *transition speed*. The average transition speed in area 18 (31°/s) was approximately twice that of area 17 (15°/s). We also found that while direction-selective neurons in area 17 lost responses to their preferred directions rapidly when speed increased, those in area 18 maintained some responsiveness even at the highest speed we tested. A spatio-temporal energy model was capable of accounting for the behavior of neurons in both areas. However, the direction-selective indexes simulated for area 18 were much higher than those observed experimentally, suggesting that direction-selective cells in area 18 exhibit some non-linear responses to moving random-dot stimuli. Altogether, our findings demonstrate that neurons with orthogonal direction preferences cooperate to cover a broad range of speeds, encoding the sequential retinotopic activations as *motion direction* at low speed, but as *motion axis* at high speed. This neural processing mechanism is common to different neurons and visual areas and therefore may serve as a fundamental principle for early visual motion processing.

## Materials and Methods

### Ethics Statement

All experimental procedures were approved by the Animal Care and Use Committee of the University of Science and Technology of China and the Institute of Neuroscience and by the local ethical review committee of the Shanghai Institutes for Biological Sciences. The cats were obtained from the institutional breeding colony.

### Animal preparation and maintenance

A total of 12 cats of either sex were used in this study. Anesthesia was achieved by intramuscular injection of ketamine hydrochloride (25 mg/kg). A tracheotomy and a venous catheterization were performed. Local anesthetic lidocaine was applied to all incisions and pressure points. The animal was transferred to stereotaxic apparatus thereafter and subsequent surgery was carried out under either gaseous anesthesia (Isoflurane 1–2% in 70∶30 N_2_O:O_2_) or pentobarbital sodium with glucose in saline (2 mg/kg/h, IV). Atropine (0.05%), gentamycin (4%), and dexamethasone (0.5%) were injected intramuscularly every 12 hours to prevent secretion, infection, and brain edema, respectively. The electrocardiogram (ECG) and pulse oxygen saturation (SpO_2_) were continuously monitored. End-tidal carbon dioxide (CO_2_) was kept around 4%. The core temperature of the animal was maintained around 38°C using a thermister controlled electric blanket. Craniotomy and duratomy were performed at Horsley-Clarke coordinates P0-P10, L0-L6 and A0-A6, L0-L6 for area 17 and 18, respectively. For intrinsic optical imaging, a stainless steel chamber was secured on the skull using dental cement, filled with silicon oil (Sigma-Aldrich, DMPS-5X), and sealed with two O-rings and a polycarbonate window [15], [21]–[22]. For single-unit recording, a plastic chamber was used and filled with 3% agar in 0.9% saline, then sealed with silicon oil or wax. During recordings, the animal was maintained by 0.5–0.7% isoflurane and muscular paralysis was obtained by intravenous infusion of gallamine triethiodide (10 mg/kg/h). Artificial respiration was achieved by a ventilator. The pupils of the cat were dilated with atropine (0.5%) and nictitating membranes were retracted with neosynephrine (1%). Zero power contact lenses were applied and correction lenses were chosen by streak retinoscopy and inserted when necessary. The retinae were back projected on a tangent screen placed 57 cm in front of the animal and the positions of the foveae were estimated from the locations of the optic disks.

### Visual stimuli

**Figure 2:**
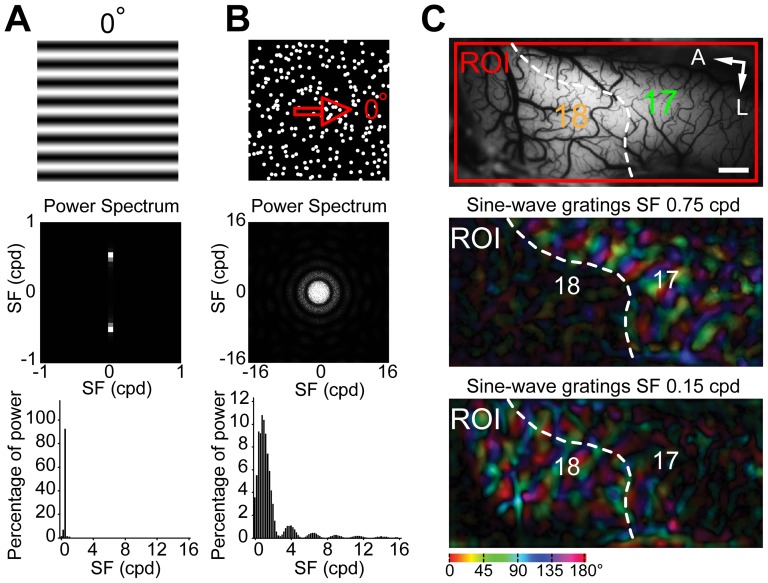
Stimuli used in this study and the discrimination of areas 17 and 18. (A–B) 0° oriented sine-wave grating with spatial frequency of 0.5 cycles per degree (cpd) (A) and random-dot field moving towards 0° direction (B). The Fourier power spectra and the power distributions along the spatial frequency dimension of the static grating and dot stimuli are shown below each stimulus diagram. Note that the power spectrum of the static random-dot field exhibits a uniform distribution in the orientation dimension. Most of the stimulus power resides at the spatial frequency of 0.5 cpd for the sine-wave grating and below 3 cpd for the random-dot field. (C) The discrimination of areas 17 and 18. An image of the cortical surface taken under 550 nm green-light illumination is displayed. The area boxed by the red rectangle was the region of interest (ROI) for optical imaging. Polar maps of orientation preference derived from sine-wave grating stimuli with spatial frequencies of 0.75 cpd and 0.15 cpd were constructed respectively, and the white broken line delimits the border between area 17 and 18 accordingly. A, Anterior; L, lateral. Scale bar: 1 mm.

A CRT monitor (Sony Trinitron Multiscan G520, 21 inches, 1280×960 pixels, 100 Hz, covering 40×30 degrees of visual angle) was placed 57 cm in front of the animal's eyes. The gamma of the monitor was corrected by using the color calibration device (ColorCAL) from Cambridge Research System. The luminance range of the screen was 0.2–87 candela per meter squared (cd/m^2^). Visual stimuli were computer-generated using custom software based on Psychtoolbox-3. Sine-wave gratings were used with 0° representing horizontal orientation (Figure 2A). The motion direction of 0° represents rightward motion (Figures 1B and 2B). In optical imaging experiments, the sine-wave gratings moved back and forth perpendicularly to their orientations for 4 s (2 s for each direction) or moved towards a single direction for 4 s to activate orientation- or direction-preference domains, respectively. To study motion signal processing, we employed random-dot field stimuli (Figure 2B) [23]–[24]. A random-dot stimulus moving along the horizontal axis was defined as having a motion axis of 0° (Figure 2B), corresponding to a spatial orientation the same as that of a horizontal bar or grating. For the activation of motion-axis preference domains, full-field random-dot stimuli were drifted back and forth for 2 s each along a given motion axis, whereas for the activation of direction-preference domains, the stimuli moved in one single direction for 4 s. Each individual dot of the random-dot field was set to maximum luminance and moved against a black background. The dots had a diameter of 0.4° and a density of ∼1.1 dots per degree squared unless otherwise stated. In addition, by doubling the dot width to 0.8° and quartering the dot density to ∼0.28 dots per degree squared, keeping the area of the screen covered by the dots constant, we tested the effect of dot size on the neuronal responses. The speeds used for the dot stimuli ranged from 1–100°/s. The spatial frequency (SF) and temporal frequency (TF) used for the sine-wave grating stimuli were between 0.1–1.4 cycles per degree (cpd) and 1–8 Hz respectively. Michelson contrast of the sine-wave gratings was 100%.

### Optical imaging and image analysis

Optical imaging was performed in five of the twelve cats. All the equipment and recording procedures used in our customized optical imaging system have been described earlier [15], [21]–[22]. We used a Dalsa Pantera 1M60 CCD camera combined with a telecentric 55 mm f2.8 video lens for simultaneously recording from both areas 17 and 18 over a certain region of interest (ROI) (Figures 1B and 2C). Within each trial there were two stimulus conditions, a pair of complementary visual stimuli, for example, sine-wave gratings with 0° and 90° orientations or random-dot stimuli with 0° and 180° directions, respectively. Under 630±10 nm red light illumination, visual responses were recorded at 16 frames per second for a period of 8 s including 1 s before stimulus onset. The interstimulus interval was 13 s. For stimuli of sine-wave gratings, data were typically averaged over 32 or 64 trials, while for random dots, data were obtained from at least 64 trials. The border between areas 17 and 18 was determined using moving sine-wave gratings with different spatial frequencies as the SF preferences in the two areas are different [25]–[27] (Figure 2C). Therefore, when a high SF was used, population responses in area 18 declined and the boundary could be delineated (Figure 2C).

For each trial, frames taken after the stimulus onset in the first 3–7 s interval were averaged. Then the average frame was subtracted and divided by a blank frame (the average response for the 1 s before stimulus start) to generate a single-condition map of reflectance change (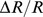). Differential orientation and motion-axis maps were created by subtracting a pair of single-condition maps activated by stimuli with orthogonal orientations or motion axes (e.g., 0°–90°). To obtain a differential direction map, we subtracted two single-condition maps elicited by a stimulus pair with opposite motion directions (e.g., 0°–180°). Orientation and direction preference maps were classically constructed using a vector summation algorithm [15], [21], [28]–[30]. We also computed direction preference maps using a vector maximum method as previously described [31]. A variability map was obtained to find pixels with large cross-trial variability and a mask was generated, based on an objectively chosen threshold [15], [21], [32]. Pixels covered by the mask were interpolated just for the clear illustration of differential and preference maps and were not used in further quantitative analysis. The images were then high-pass filtered (1.1–1.2 mm in diameter) and smoothed (85–323 μm in diameter) by circular averaging filters. To find significant direction-selective pixels, we adopted a recent method to calculate direction preference p-value map for sine-wave grating stimuli [33]. A mask was generated to exclude pixels with p>0.05. We also used this alternative mask in the comparison of different direction preference maps. As results obtained using the masks derived from the p-value map and the variability map were consistent, we only presented results computed using the mask derived from the variability map. Response profile analysis [12], [15], [21], [32] was performed to extract the orientations represented by the differential orientation or motion-axis maps as well as the directions represented by the differential direction maps. Differential orientation or motion-axis map (e.g., 0°–90°) was divided up into 12 equally spaced orientation bins (from 0° to 180° in 15° step) that were derived from the orientation preference map. Similarly, a differential direction map (e.g., 0°–180°) was divided up into 12 equally spaced direction bins (from 0° to 360° in 30° step) that were derived from the direction preference map generated by sine-wave gratings. Each orientation/direction bin contains the cortical locations preferring the corresponding orientation/direction. Therefore, these processes enable the pixels of the differential maps to be grouped based on their orientation/direction preferences. The signs of 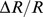 values in the differential map were reversed, so that pixels responsive to the first condition (e.g., 0° orientation, 0° motion axis, or 0° direction) had positive values while pixels responsive to the second condition (e.g., 90° orientation, 90° motion axis, or 180° direction) obtained negative values, then the mean was subtracted from each pixel. The 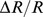 values of pixels within each of the orientation/direction bins were averaged to give a measurement of response strength of cortical locations preferring that orientation/direction before a complete response profile, across all bins, was produced. The response profile was fitted with a full cycle of a cosine function as follows:
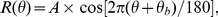
(1)where R is the response strength as a function of θ/2θ the equally spaced orientations/directions. A and θ_b_/2θ_b_ are the parameters to be fitted, corresponding to maximum response strength and orientation/direction best represented by the differential map respectively.

### In vivo single-unit recording and data analysis

As a rule but not always, we performed intrinsic optical imaging before single-cell recordings. Glass-coated and epoxylite-insulated (FHC Inc., Maine, USA) tungsten microelectrodes (3–5 MΩ) were used to record extracellular electric signals (Figure 1B). The electrode was advanced vertically via a hydraulic micromanipulator (Narishige, Tokyo, Japan). To record neurons in area 17, we made penetrations at Horsley-Clarke coordinates P1-P6, L0-L2.5 according to the geometric structure of the exposed striate cortex on the surface, while we targeted A1–A6, L2–L5 for studying neurons in area 18. Recorded analogue signals were amplified (Molecular Devices, California, USA or Dagan, Minnesota, USA) and digitized using Power 1401 (Cambridge Electronic Design, Cambridge, England) under spike2 software control or using a data acquisition board (National Instruments, Texas, USA) controlled by customized programs operated in the MATLAB (Mathworks, Massachusetts, USA) environment. The spikes were offline sorted using the software of spike2 Version6 (Cambridge Electronic Design, Cambridge, England).

We searched for cells using full-field sine-wave gratings. After a cell was encountered and spikes were well isolated, the receptive field was determined via a hand-held bar. Stimuli were presented to the dominant eye while the other eye was occluded. Either eye was stimulated if the two eyes responded equally well. Stimuli were displayed in a circular window covering the receptive field of the cell. Spatial and temporal frequency preferences were determined using drifting sine-wave gratings. Direction tuning curves for gratings were measured using optimal spatial and temporal frequencies. The cells were classified as simple or complex based on classical criteria of the ratio between fundamental Fourier components and DC components (F1/F0) of the responses measured with preferred spatial frequencies [34]–[38]. Some of the cells were unclassified as we tested them with the random-dot stimuli first and the cells were lost afterwards. Direction selectivity to random-dot field was examined with 12 equally spaced directions (0°–360°) and 8–9 different speeds (selected from speeds of 1, 4, 5, 7, 10, 15, 20, 30, 50, 75, 80, 100°/s). Stimuli with different combinations of direction and speed were displayed pseudo-randomly for a period of 1–2 s and each of the stimuli was presented 10 times. A static random-dot field was shown for 1–3 s between different moving conditions and the responses to the static stimuli were used as a measurement of spontaneous activity of the cell. The mean firing rate during the period of stimulus motion was used to generate the direction tuning curves. Cells with low firing rates (<4 spikes/s) and large cross-trial variability to grating or random-dot stimuli were excluded from further analysis. A total of 46 cells in area 17 and 41 cells in area 18, the receptive fields of which lay within 12° of eccentricity, were thoroughly studied with random-dot stimuli.

A vector summation method was used to quantitatively characterize the direction tuning curves for the ease of comparison with optical imaging results as follows: 
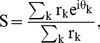
(2)where 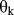 and 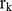 are the direction of motion and mean firing rate, respectively. The complex phase and amplitude of the resultant S represent the preferred direction (PD) and direction selective index (DSI), respectively. DSI varies between 0, for a cell that responds equally to all directions, and 1, for a cell that only responds to a single direction. Preferred motion axis (PA) and motion-axis selective index (ASI) for random-dot stimuli were computed in a similar manner, except that 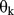 was replaced by 2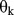 in the above equation and the resultant phase halved. Preferred orientation (PO) and orientation selective index (OSI) to sine-wave grating were also calculated by substituting 2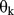 for 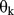, while π was added to the resultant phase before it was halved as the moving directions of gratings were always perpendicular to the orientations. Cells were classified into orientation- (OSI>0.2 and DSI<0.2) and direction- (DSI>0.2) selective categories based on their responses to sine-wave grating stimuli [39]–[40]. We also classified cells into direction-selective category using directionality index adopted from a previous study [41]. As results were nearly identical based on different classification criteria, we only presented the results derived from DSI and OSI.

### Monitor fidelity validation

As the light emitted from the fluorescent material on the screen of the CRT monitor needs a certain time to completely die out, the residual luminance of the white dots in one frame might persist in the next frame and be visually detected. In this situation, physical speed lines will appear on the monitor when the dots move especially at high speed, which could be detrimental for studying neural processing of dot motion. To test whether the monitor could cause a significant artifact, we took pictures of the screen using a SLR camera (Canon EOS 5D Mark II) under the experimental condition. The exposure time and ISO were set to 1/4000 s and 1600 respectively. Through quantifying the intensity of the dots in the pictures, we found that after quenching for 10 ms (refresh rate 100 Hz), the residual luminance (<0.28 cd/m^2^) of the dots was more than 200 folds lower than the luminance (∼87 cd/m^2^) of dots just emerged in the next frame and only slightly higher than the black-background luminance (∼0.2 cd/m^2^) (Figure S1). To further address whether the residual luminance was negligible, we increased the background luminance of the random-dot field stimuli to 15 cd/m^2^ (gray background). The contrast between the residual dots and the gray background will be less than 1% and would exceed the physiological contrast sensitivity of cat [42]–[43]. We performed control experiments in another two cats using random-dot stimuli with the gray background. The results obtained with the gray and black backgrounds were consistent (see examples in Figures S2–3), validating the stimuli used in our study.

### Model simulation

We used a simple spatio-temporal energy based model to simulate our optical imaging and electrophysiological results. The model structure has been described in previous studies [13]–[15], but was slightly modified in the present study. Neurons in areas 17 and 18 were modeled as three separable linear filters using Gaussian functions as follows: 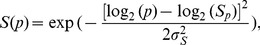
(3)

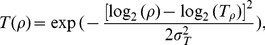
(4)


Where 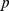 and 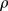 are the spatial and temporal frequency coordinates. As the temporal frequency filter was symmetrical in frequency space, for the ease of calculation, only the positive half of the temporal frequency coordinate was considered. 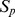 and 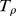 are the preferred spatial and temporal frequencies; 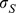 and 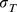 are proportional to the spatial and temporal bandwidth in octaves. The orientation tuning curve (Eq. 5) was defined as a sum of Gaussian functions of two direction filters for opposite directions, 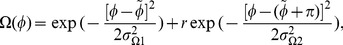
(5)where 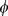 is the direction coordinates; 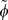 is the peak direction; 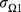 and 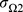 are the characteristic widths of the two individual direction filters. For simplicity, we set 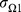 = 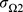. 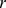 is a weight (ranging from 0 to 1) that determines the relative contribution of the direction filter opposite to the preferred one. We used 0 and 1 for the simulation, with 0 representing a highly direction-selective cell, and 1, a highly orientation-selective cell. For a given stimulus, neuronal or population responses were computed as the integral over all spatiotemporal frequencies and orientations of the stimulus as follows: 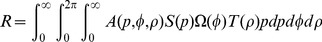
(6)where 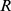 and 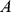 are the response and Fourier transformed stimulus respectively.

To model population responses recorded optically, we simplified the problem by assuming that neuronal populations have the same spatial and temporal tuning properties in the visual cortex of areas 17 and 18. We used the value 1 for the parameter 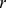 of the orientation filter (Eq. 5) to simulate population responses to orientation and axis of motion and the value 0 was used to simulate population responses to direction. As in the optical imaging data, responses to stimuli with orthogonal orientations, orthogonal motion axes, or opposite directions were subtracted to obtain modeled response profiles for comparison with optical imaging results. The modeled response profile was also fitted with a cosine function (Eq. 1). To simulate single-unit recording results, values of 0 and 1 were used for the parameter 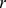 in Eq. 5 for modeling responses from highly direction- and orientation-selective cells, respectively. Responses to different moving directions of sine-wave gratings and random-dot stimuli were computed and the vector summation method (Eq. 2) was used to characterize the response curves. For the simulation results, we averaged 32 trials as presented on the monitor during experiments (we only analyzed a 15°×15° subregion to reduce computation time; results were well approximated by this reduction). The parameters for the three filters of the model were adopted from previous well-known studies and our single-unit data in cat areas 17 and 18 [26], [44]–[54] and the bandwidths were converted from reported bandwidths to fit into the Gaussian functions (Table 1).

**Table 1:** Parameters used for the model simulation.

Visual Area	Preferred SF ( 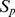 )	SF Bandwidth ( 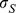 )	Preferred TF ( 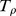 )	TF Bandwidth ( 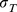 )	Direction Bandwidth ( 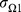 )
**17**	0.44 cpd	0.64 cpd	2.23 Hz	1.36 Hz	15.3°
**18**	0.2 cpd	0.63 cpd	5 Hz	1.27 Hz	21.2°

SF, spatial frequency; TF, temporal frequency.

## Results

### Transition speed in area 18 is much higher than that in area 17

**Figure 3:**
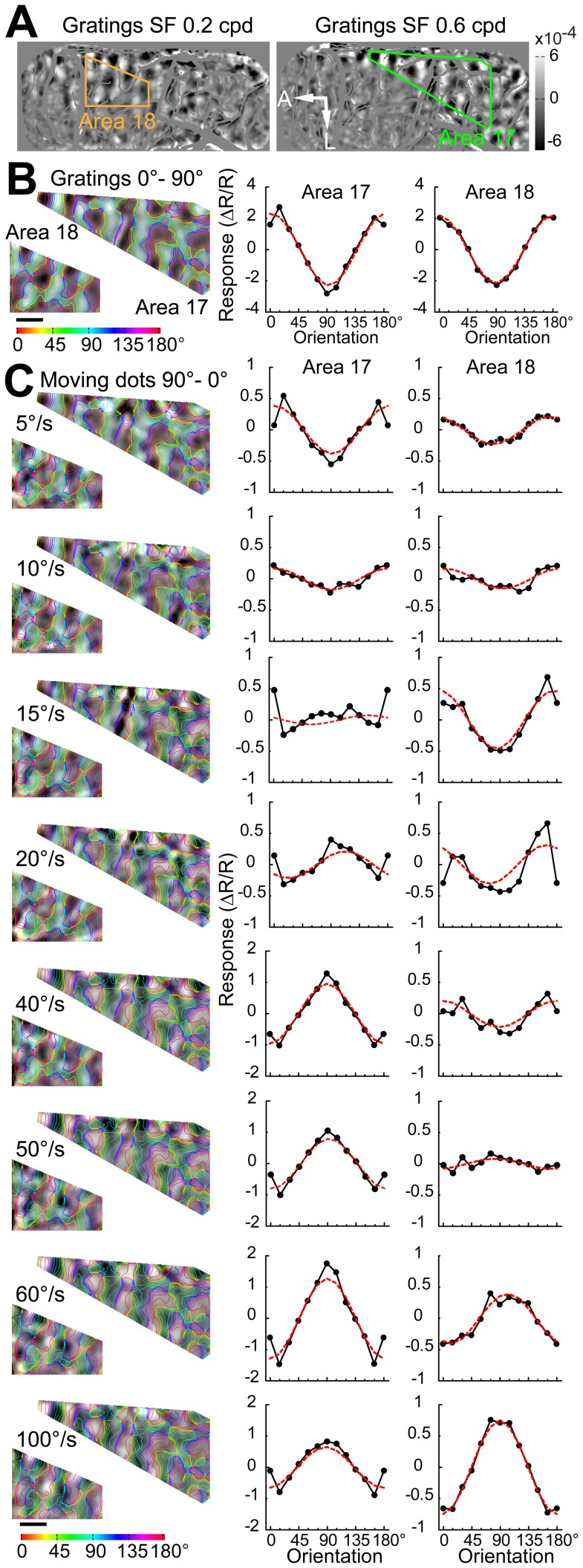
Differential population responses to different motion axes. (A) Differential orientation maps generated by drifting sine-wave gratings with SFs of 0.6 cpd and 0.2 cpd for driving areas 17 and 18, respectively. Blood vessels and marginal regions were masked gray on the maps. Responses of selected regions from areas 17 and 18 (green and orange outlines) were analyzed in (B) and (C). A, Anterior; L, lateral. (B) Differential orientation maps (0°–90°) and the corresponding results of response profile analysis acquired using sine-wave grating stimuli. (C) Differential motion-axis maps (90°–0°) and the corresponding results of response profile analysis obtained by moving random-dot stimuli with different speeds. Colored iso-orientation contours were derived from the orientation preference map for sine-wave grating stimuli and were superimposed on the gray images. The red curves in the plots represent the best fitting cosine functions. The values of the ordinates correspond to the recorded responses (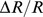) × 10^4^. Note that the scales of y axes are different between low and high speeds. Scale bar: 1 mm.

**Figure 4:**
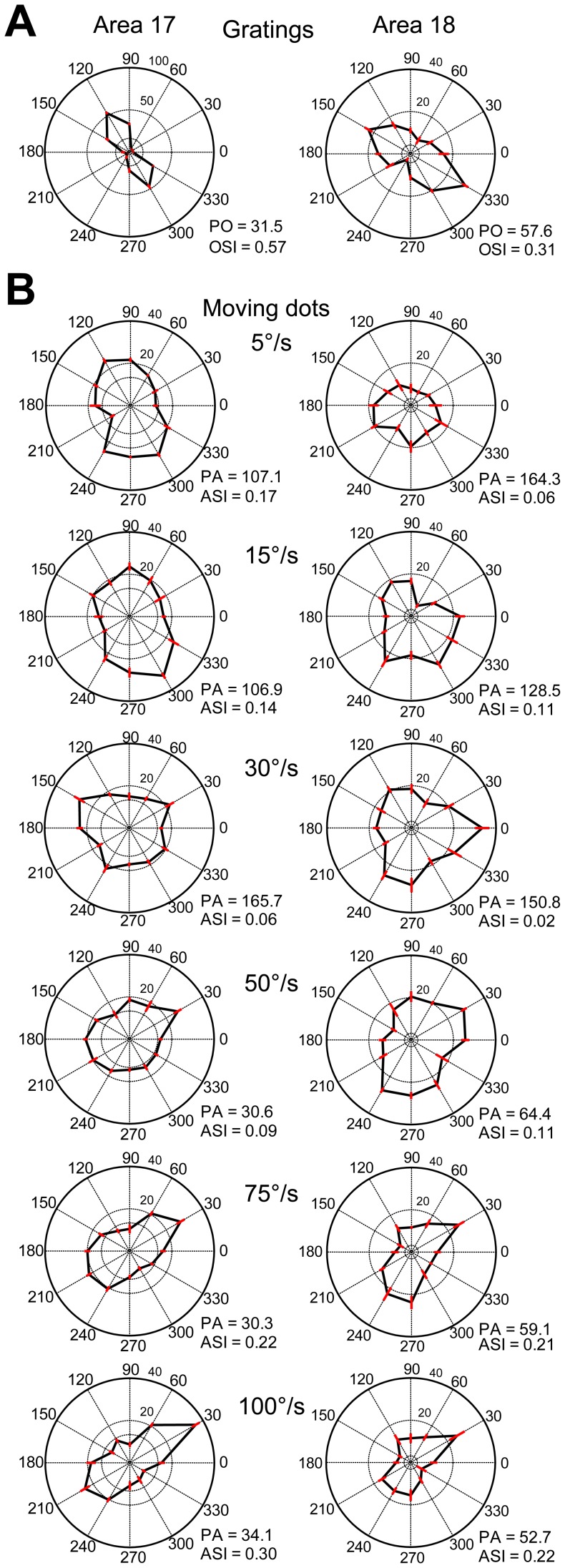
The responses of two orientation-selective cells in areas 17 and 18 to different moving directions and speeds of random dots. (A) Typical polar plots of direction tunings of orientation-selective cells generated using sine-wave grating stimuli. PO, preferred orientation; OSI, orientation-selective index. N = 5 trials for both cells. (B) Polar plots of direction tunings acquired by using random-dot stimuli moving at different speeds. PA, preferred motion axis; ASI, motion-axis selective index. N = 10 trials for both cells. The spontaneous firing rates of the cells are 13 spikes/s and 3 spikes/s in areas 17 and 18, respectively. Error bars represent SEM.

**Figure 5:**
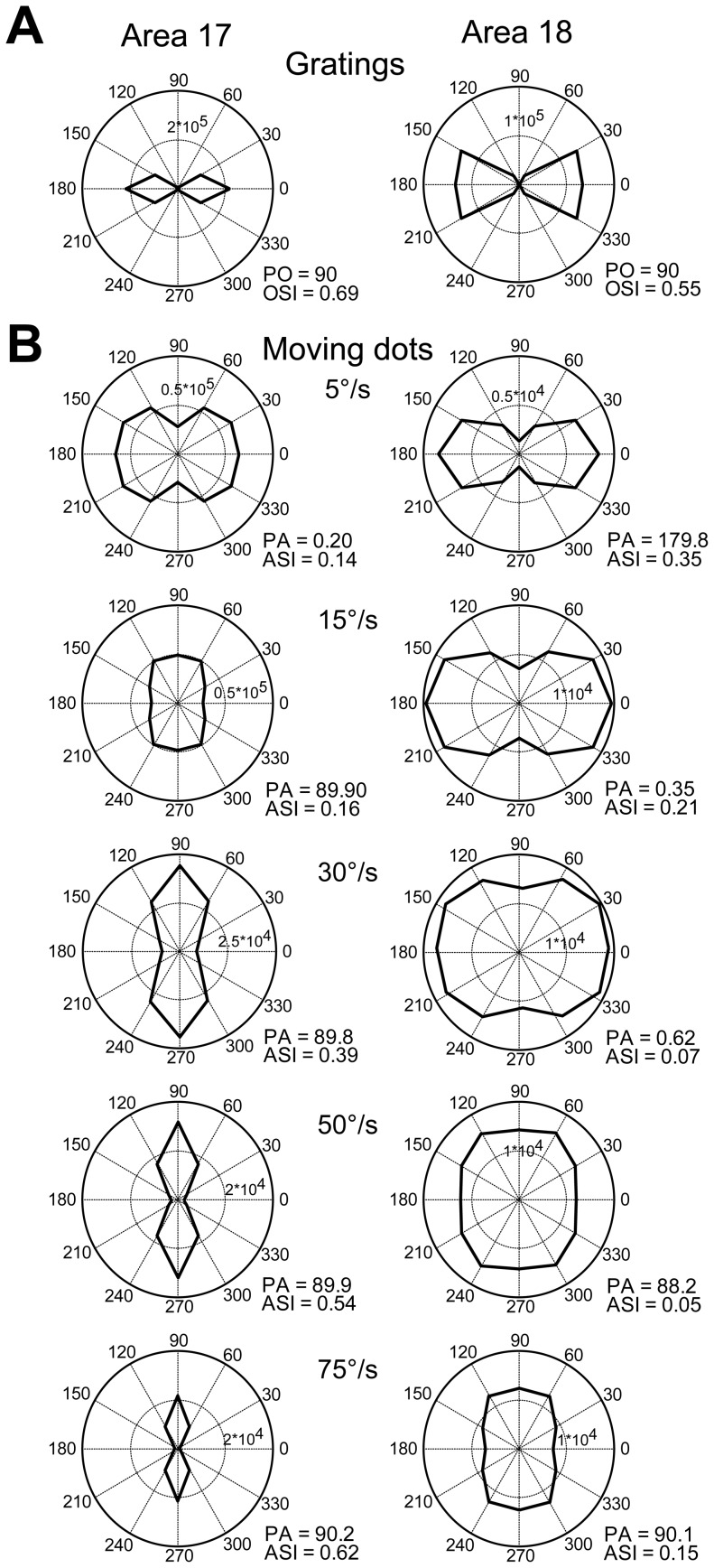
The simulated responses of orientation-selective cells to moving random-dot stimuli with different speeds. (A) Polar plots of direction tunings of simulated neuronal responses to drifting sine-wave gratings. PO, preferred orientation; OSI, orientation-selective index. (B) Polar plots of direction tunings of simulated neuronal responses to random-dot stimuli moving at different speeds. PA, preferred motion axis; ASI, motion-axis selective index.

Consistent with previous optical imaging study in macaque V1, V2, and V4 and electrophysiological study on neuronal populations in cat area 17 [10]–[11], [15], we found that population responses of orientation-selective neurons in cat area 18 also devoted specifically to encode motion-axis information when dots moved above certain transition speeds (Figure 3). This is because when above transition speeds, moving dots created a motion streak parallel to the preferred orientation of the orientation-selective neurons. However, the transition speed in area 18 was drastically higher than that in areas 17 in all cases; the transition speed in this case was about 50°/s for area 18 and 15°/s for area 17, respectively (Figure 3C). The population responses were confirmed by single-unit recordings in both areas (see examples in Figure 4). In addition to the prediction of population responses in ferret area 17 as previously reported [13]–[14], the spatio-temporal energy model also reproduced the single-cell behavior of orientation-selective neurons at various speeds in both areas 17 and 18 (Figure 5). These results revealed that at high speed orientation-selective neurons in area 18 also encode motion axes that are parallel to their preferred orientations, but the transition speed in area 18 is higher than that in area 17.

### Population responses to the moving direction of random dots at different speeds

**Figure 6:**
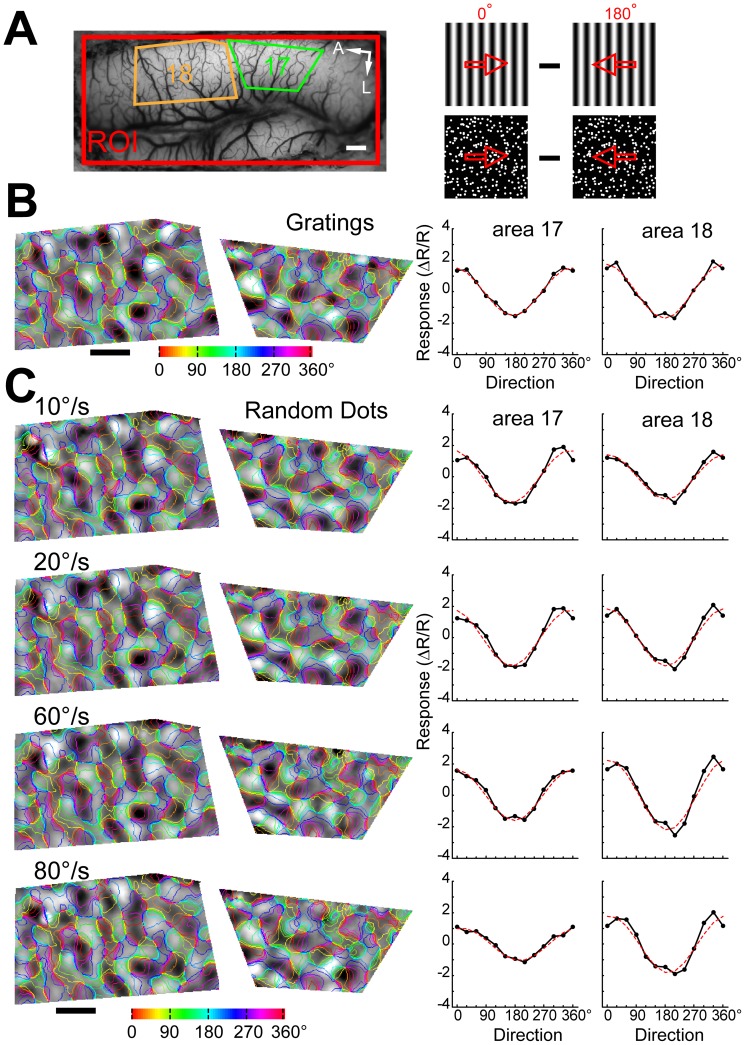
Differential population responses to different directions of motion. (A) Image of the cortical vasculature and schematic diagrams of the stimuli used for driving direction-selective responses. The irregular green and orange polygons define the selected regions of areas 17 and 18 for further analysis, respectively. Red arrows in the stimulus diagrams indicate the moving directions. A, Anterior; L, lateral. Scale bar: 1 mm. (B) Differential direction maps (0°–180°) and the corresponding results of response profile analysis acquired using stimuli of drifting sine-wave gratings. (C) Differential direction maps (0°–180°) and the corresponding results of response profile analysis obtained by moving random-dot stimuli with different speeds. Colored iso-direction contours were derived from the direction preference map for sine-wave grating stimuli and were superimposed on the gray images. The red curves in the plots represent the best fitting cosine functions. The values of the ordinates correspond to the recorded responses (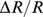) × 10^4^.

**Figure 7:**
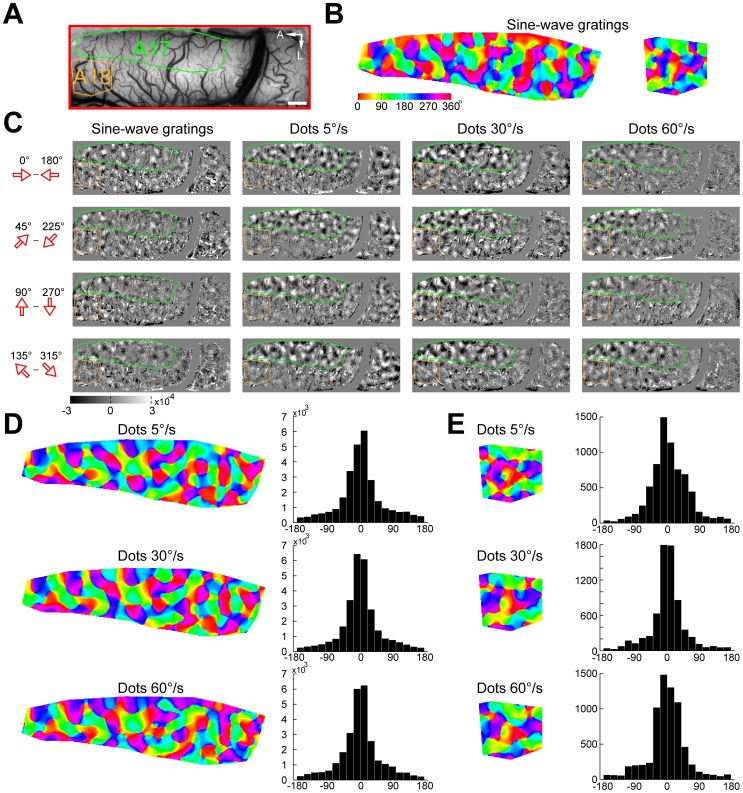
Direction preference maps calculated by vector summation were not changed with moving speed of random-dot stimuli. (A) Image of the cortical vasculature. The green and orange polygons indicate regions of areas 17 and 18 selected for analysis, respectively. A, Anterior; L, lateral. Scale bar: 1 mm. (B) Direction preference maps calculated by vector summation acquired using sine-wave grating stimuli in areas 17 and 18. Hues in the color maps indicate direction preferences. (C) Differential direction maps activated by four different pairs of directions of moving sine-wave gratings and random dots with different speeds. (D–E) Direction preference maps calculated by vector summation acquired using random-dot stimuli with different speeds in areas 17 (D) and 18 (E). Angular differences between direction preference maps of sine-wave grating and of random-dot stimuli with different speeds are shown in the histograms. Computed from the angular-difference histograms of 5°/s, 30°/s, and 60°/s,the percentages of pixels with angular difference between −60° and 60° amount to 73%, 78%, and 75% in area 17 (D) and 78%, 82%, and 80% in area 18 (E), respectively.

**Figure 8:**
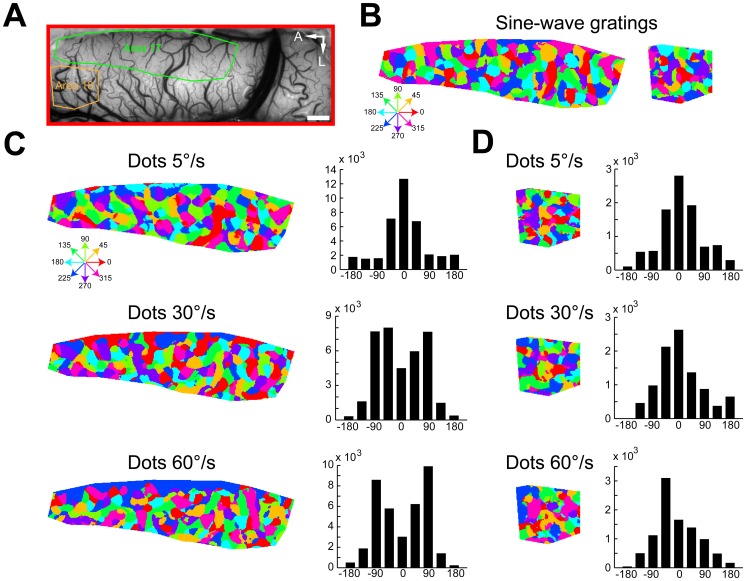
Direction preference maps calculated by vector maximum were changed with moving speed of random-dot stimuli. (A) Image of the cortical vasculature. The green and orange polygons indicate regions of areas 17 and 18 selected for analysis, respectively. Note that the selected regions are the same regions analyzed in Figure 7. A, Anterior; L, lateral. Scale bar: 1 mm. (B) Direction preference maps calculated by vector maximum acquired using sine-wave grating stimuli in areas 17 and 18. Hues in the color maps indicate direction preferences. (C–D) Direction preference maps calculated by vector maximum acquired using random-dot stimuli with different speeds in areas 17 (C) and 18 (D). Angular differences between direction preference maps of sine-wave grating and of random-dot stimuli with different speeds are shown in the histograms. Computed from the angular-difference histograms of 5°/s, 30°/s, and 60°/s, the percentages of pixels with angular difference between −45° and 45° amount to 71%, 49%, and 40% in area 17 (C) and 69%, 64%, and 65% in area 18 (D), respectively.

Differential direction maps were generated using either pairs of sine-wave gratings or random-dot stimuli moving in opposite directions (e.g., 0°–180°) at different speeds (Figure 6A). Sine-wave grating and random-dot stimuli all activated direction-responsive domains in both areas 17 and 18 (Figure 6B–C). The response patterns to moving dots of different speeds were precisely in register with those to sine-wave gratings. The fitted directions derived from the response profile analysis were all near 0° (peaks in Figure 6C). Results from another cat for four different pairs of stimulus directions are presented in Figure 7C. We calculated direction preference maps from single condition maps of eight different directions (0°–360° in 45° steps) (Figure 7) using vector summation [15], [55]. We compared the preference maps for sine-wave grating stimuli with those for random-dot stimuli of different speeds (Figure 7B, D–E). The angular difference histograms constructed by subtracting preference maps from areas 17 and 18 all showed a single peak around 0° (Figure 7D–E). This gave rise to a rather surprising result as direction preference maps are not changed with speed. However, it has been noticed that the vector summation method cannot faithfully reveal the actual preferred direction in some conditions, such as responses in preferred and non-preferred directions are nearly equal in magnitude [56]. Instead, a vector maximum method, which represents the ‘electrophysiologist's ear’ approach, was proposed as a better method for decoding direction preference from intrinsic signals [56]. We speculated that the above result might be due to the method used for calculating direction preference. Therefore, we switched to the vector maximum method to regenerate direction preference maps. This time, we found that the direction preferences of maximum population responses indeed changed with speed in both areas (Figure 8). The change in the preferred direction can be, for example, as much as 90° at 60°/s in area 17 (Figure 8C). These results suggest that the vector maximum rather than the vector summation method shall be used to decode the direction preference of population responses recorded by intrinsic optical imaging, particularly when random-dot stimuli are employed and the moving speed is high.

**Figure 9:**
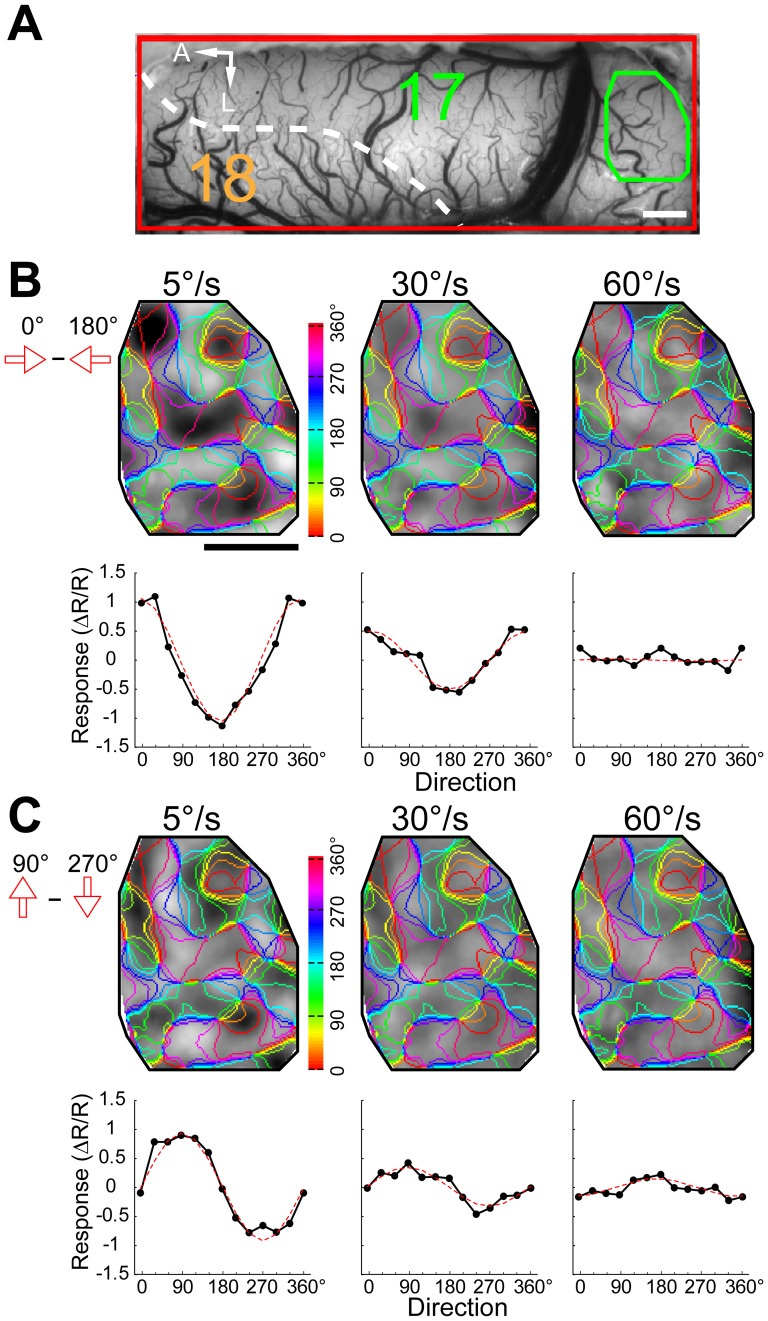
Direction differential maps and response profile analyses from a posterior part of area 17. (A) Image of the cortical vasculature. The white broken line delimits the border between area 17 and 18. The green polygon indicates the posterior part of area 17 selected for analysis. A, Anterior; L, lateral. Scale bar: 1 mm. (B–C) Differential direction maps (0°–180°, B; 90°–270°, C) and the corresponding results of response profile analysis obtained by moving random-dot stimuli with different speeds. Colored iso-direction contours were derived from the direction preference map for sine-wave gratings and were superimposed on the gray images. The red curves in the plots represent the best fitting cosine functions. The values of the ordinates correspond to the recorded responses (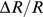) × 10^4^.

Furthermore, we found that the response domains in the posterior part of area 17 gradually disappeared with the increase of speed and no longer demonstrated a clear pattern at 60°/s (Figure 9). This observation was confirmed by response profile analyses. As neurons in the posterior part of area 17 have small eccentricities corresponding to the central visual field and prefer high SF and low TF [54], [57]–[58], a speed of 60°/s may reduce or abolish the response to the preferred directions in this portion of area 17. Therefore, this result suggests that the response strengths to the preferred directions of neurons greatly reduce at high speed.

**Figure 10:**
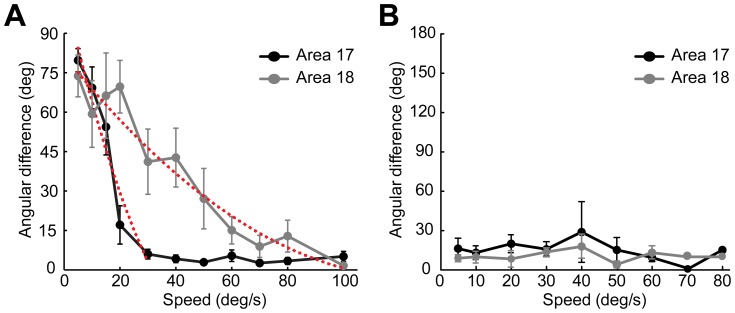
Summary of the population responses to random-dot motion with different speeds in areas 17 and 18. (A) Angular differences between the fitted orientations of differential motion-axis maps and the motion axes of the moving-dot stimuli were plotted against speed. The red dotted curves represent the quadratic polynomial fits. (B) Angular differences between the fitted directions of differential direction maps and the moving directions of the random-dot stimuli. Results were calculated through all speeds tested and averaged across 5 cats studied using intrinsic optical imaging. Error bars represent SEM.

Finally, we fitted all the curves from response profile analysis to summarize the characteristics of differential population responses in both areas 17 and 18 across all cats studied (Figure 10). The angular difference between the motion axis of the random-dot stimuli used and the fitted orientation of the differential motion-axis responses was calculated for all speeds tested. We found that the angular differences of population responses in both areas were significantly changed with speed (p<0.01, two-way ANOVA) (Figure 10A). The angular differences decreased close to 0 degree when motion speeds were above 20°/s and 60°/s, respectively, for areas 17 and 18 (Figure 10A). These results demonstrate that the preferences of population responses activated by the motion axis of moving dots change by 90° in orientation from low to high speed. To obtain transition speed for the average population results (Figure 10A), we fitted the curve of area 18 with a quadratic polynomial function (R^2^ = 0.93). For area 17, only the first five points were fitted (R^2^ = 0.94), because after 40°/s the angular differences reached a plateau close to 0 degree. The computed speeds, at which a 45° angular difference was reached in the fitted curves, were 15°/s for area 17 and 31°/s for area 18. The angular difference between the direction of the moving random-dot stimuli used and the fitted direction derived from the differential direction map was also computed (Figure 10B). We found the angular differences for direction were neither significantly changed with speed (p = 0.81, two-way ANOVA) nor with visual area (p = 0.27, two-way ANOVA). The average angular differences in direction were all below 30° in both areas across all speeds tested, suggesting that a similar pattern of differential direction maps is activated at different speeds [15]. This result was found to be caused by the subtraction method used for generating the differential direction maps and was next addressed by single-unit recording of direction-selective cells.

### Responses of single direction-selective neurons to random-dot stimuli with different moving speeds

We performed single-unit recordings in both areas 17 and 18 to specifically address the following two questions: how do single direction-selective neurons in early visual cortices respond to random-dot stimuli moving at different speeds and how are these neuronal responses correlated with intrinsic signals of population activities?

**Figure 11:**
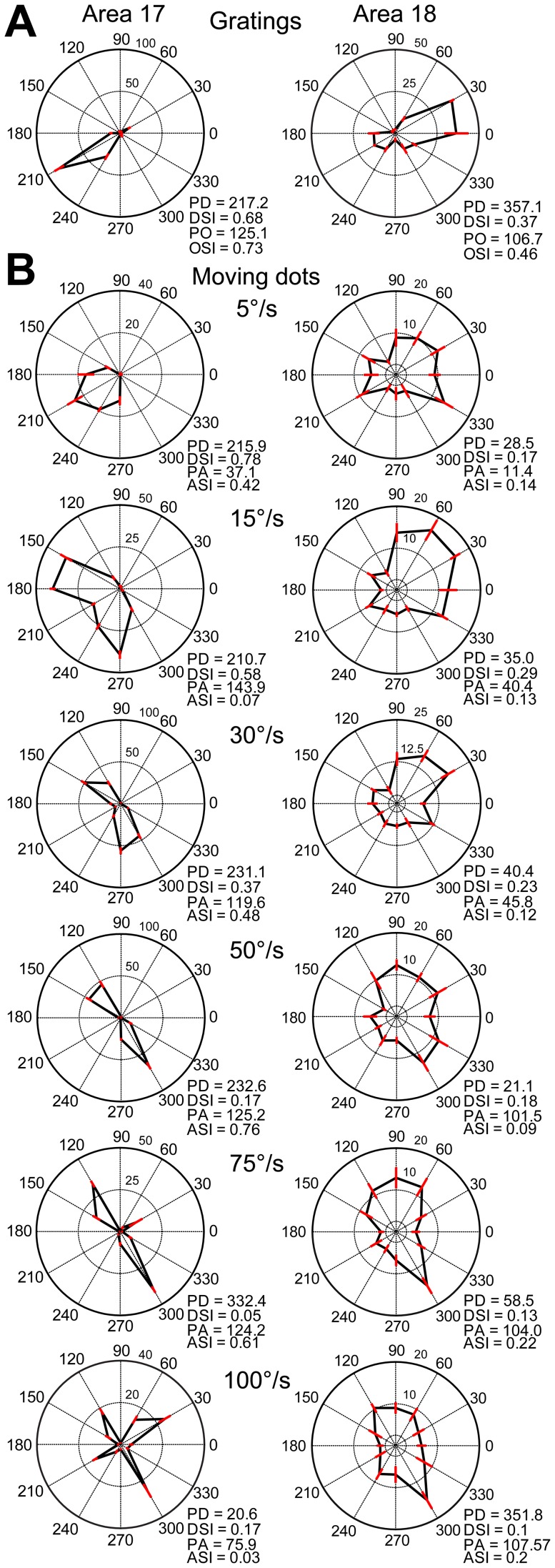
Responses of two direction-selective cells in areas 17 and 18 to different moving directions and speeds of random dots. (A) Typical polar plots of direction tunings of direction-selective cells generated using sine-wave grating stimuli. PD, preferred direction; DSI, direction selective index; PO, preferred orientation; OSI, orientation selective index. N = 6 and 5 trials for the cells in areas 17 and 18 respectively. (B) Polar plots of direction tunings acquired by using random-dot stimuli moving at different speeds. PA, preferred motion axis; ASI, motion-axis selective index. N = 10 trials for both cells. The spontaneous firing rates of the cells are 0 spikes/s and 2 spikes/s in areas 17 and 18 respectively. Error bars represent SEM.

Responses from two direction-selective neurons to moving grating stimuli are shown in Figure 11A. When stimulated with random dots moving at a low speed (5°/s), the widths of the direction tuning curves of these two neurons were broader than those with grating stimuli in both areas 17 and 18 (Figure 11B). The preferred motion axes at low speeds were almost perpendicular to the preferred orientations for grating stimuli (Figure 11A). However, the single-peaked direction tuning curves changed into a bimodal shape when speed increased. The newly developed two peaks were some distance away on either side of the original single peak at low speed (5°/s) and became more widely separated from each other when speed increased (Figure 11B). This observation was more conspicuous in the neuron of area 17 (left column of Figure 11B). Furthermore, the preferred motion axes at high speeds became perpendicular to those at low speeds in both areas. A speed of 100°/s generated a four-lobed response profile with low firing rates in area 17 (Figure 11B) and the resulting value was not appropriate to be used for the comparison. This kind of four-lobed response at very high speeds was rare in our samples. The moving dots may need to pass through a specific zone of the receptive field to produce the four-lobed response pattern [59]–[60], while the dots in our stimuli were positioned randomly. Interestingly, when calculated using vector summation, the preferred directions of both neurons to random-dot stimuli hardly changed with speed when compared with those to grating stimuli (less than 16° and 44° for the neurons in areas 17 and 18, respectively, below speed of 75°/s) (Figure 11). This confirms the population results on direction preference maps as the independency of preferred direction with speed occurs only when using the vector-summation method (Figure 7).

**Figure 12:**
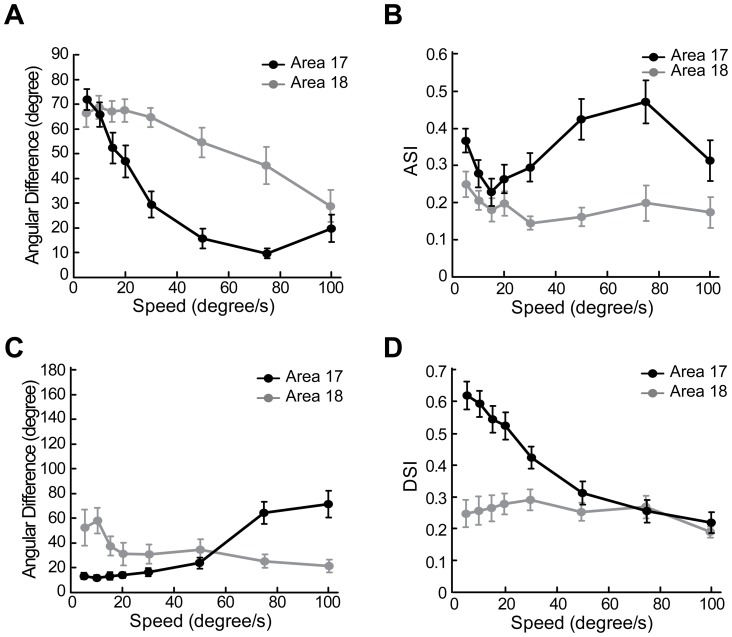
Summary of the response properties of direction-selective neurons to random-dot motion at different speeds. (A) Angular differences between the preferred orientations for sine-wave gratings and the preferred motion axes for moving random dots. (B) The influence of speed on motion-axis selective index (ASI). (C) Angular differences between the preferred directions for moving sine-wave gratings and for moving random dots. (D) The influence of speed on direction-selective index (DSI). Error bars represent SEM.

**Figure 13:**
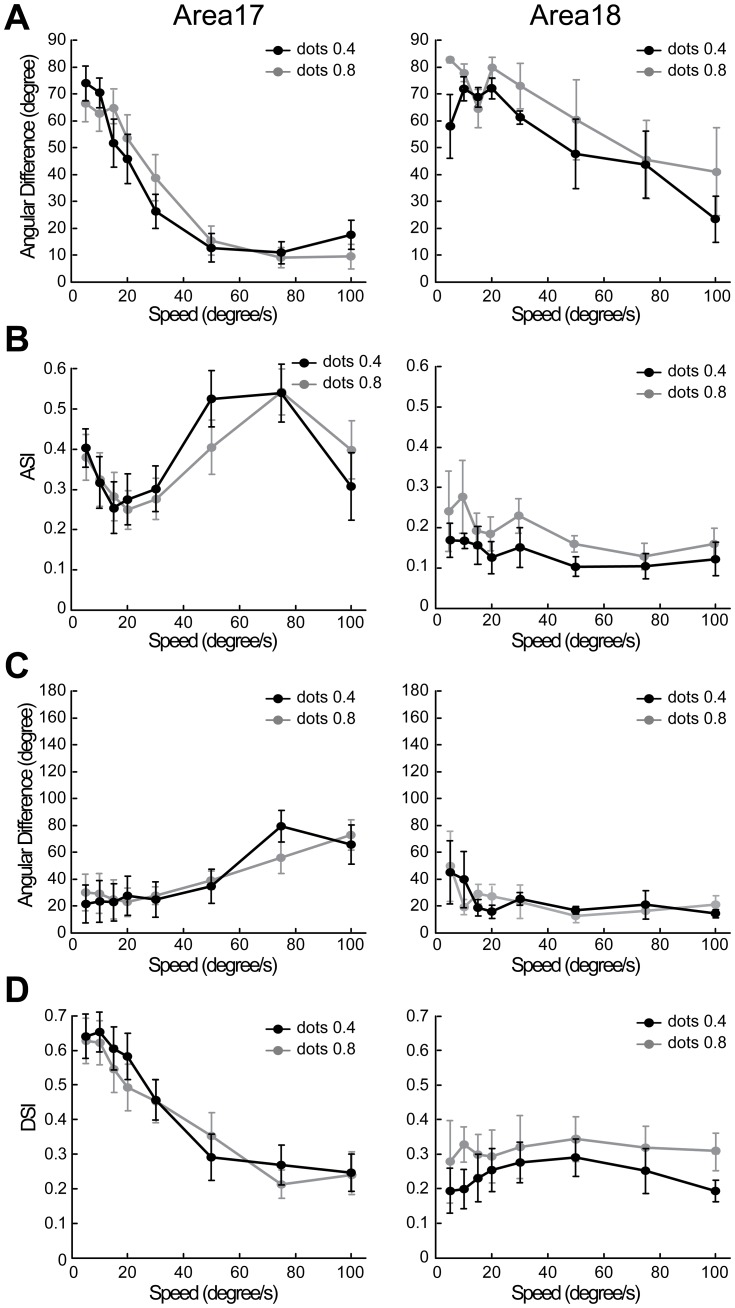
Response properties of direction-selective neurons to moving dots with size of 0.4° and 0.8° were similar. (A) Angular differences between the preferred orientations for sine-wave gratings and the preferred motion axes for moving random dots. (B) The influence of dot size on motion-axis selective index (ASI). (C) Angular differences between the preferred directions for moving sine-wave gratings and for moving random dots. (D) The influence of dot size on direction-selective index (DSI). Error bars represent SEM.

To quantitatively evaluate the changes in neuronal behavior for a direct comparison with optical imaging results, we plotted the angular differences between preferred motion axes and preferred orientations against speed (Figure 12A). Results were derived from all direction-selective neurons recorded (23 in area 17, 23 in area 18). Similar with optical imaging results, the angular differences of neuronal responses in both areas were significantly changed with speed (p<0.001, two-way ANOVA). Angular differences of nearly 90° were found in both areas when speeds were low, but the slope of the curve for area 17 was steeper than that for area 18 (Figure 12A). The angular differences were significantly higher in area 18 than those in area 17 in the range of 30–75°/s (p<0.05, two-way ANOVA, post hoc Bonferroni test). In addition, we found the motion-axis selective indexes first decreased then increased with the increase of speed in both areas (Figure 12B). This tendency was more apparent in area 17 than that in area 18. Furthermore, the angular differences between preferred directions (calculated with vector summation) for gratings and for dots moving at different speeds were calculated (Figure 12C). The resulting angular differences were below 30° in area 18 along the increase of speed. Noting that, the angular differences became larger than 60° in area 17 at 75 and 100°/s. This is mainly because at these high speeds direction-selective neurons in area 17 exhibited very weak direction selectivity, as reflected in the direction-selective indexes (DSIs) (Figure 12D). Therefore, the preferred direction computed mainly reflected the responses to the motion axis rather than the direction. Compared with low speed, the neuronal responses to moving dots at high speed also became more variable and weaker in area 17, leading to larger variations in the computed preferred directions. The DSIs for the low-speed range of 5–20°/s were significantly lower in area 18 compared with those in area 17 (p<0.05, two-way ANOVA, post hoc Bonferroni test). In addition, the increase in speed had less effect on the DSIs of area 18 compared to area 17 (Figure 12D). This was mainly due to neurons in area 18 showed weak responses with large variability to dots moving at low speeds. To study the influence of dot size on the response property of neurons, we tested in some neurons (11 in area 17, 5 in area 18) using moving dots with sizes of both 0.4° and 0.8°. By this doubling of dot size, we observed almost no change of response properties in either area 17 or 18 (Figure 13). Altogether, these single-cell recordings in both areas demonstrate that when motion speed is high, neurons with a direction preference orthogonal to the motion direction become dominant and encode the motion axis signal parallel to the direction of motion as a spatial orientation code. These results also suggest that in the early visual brain, different groups of direction-selective neurons with orthogonal direction preferences are sequentially engaged for the processing of direction at low speed, but motion axis at high speed.

### No differences between simple and complex cells for the encoding of motion streak

**Figure 14:**
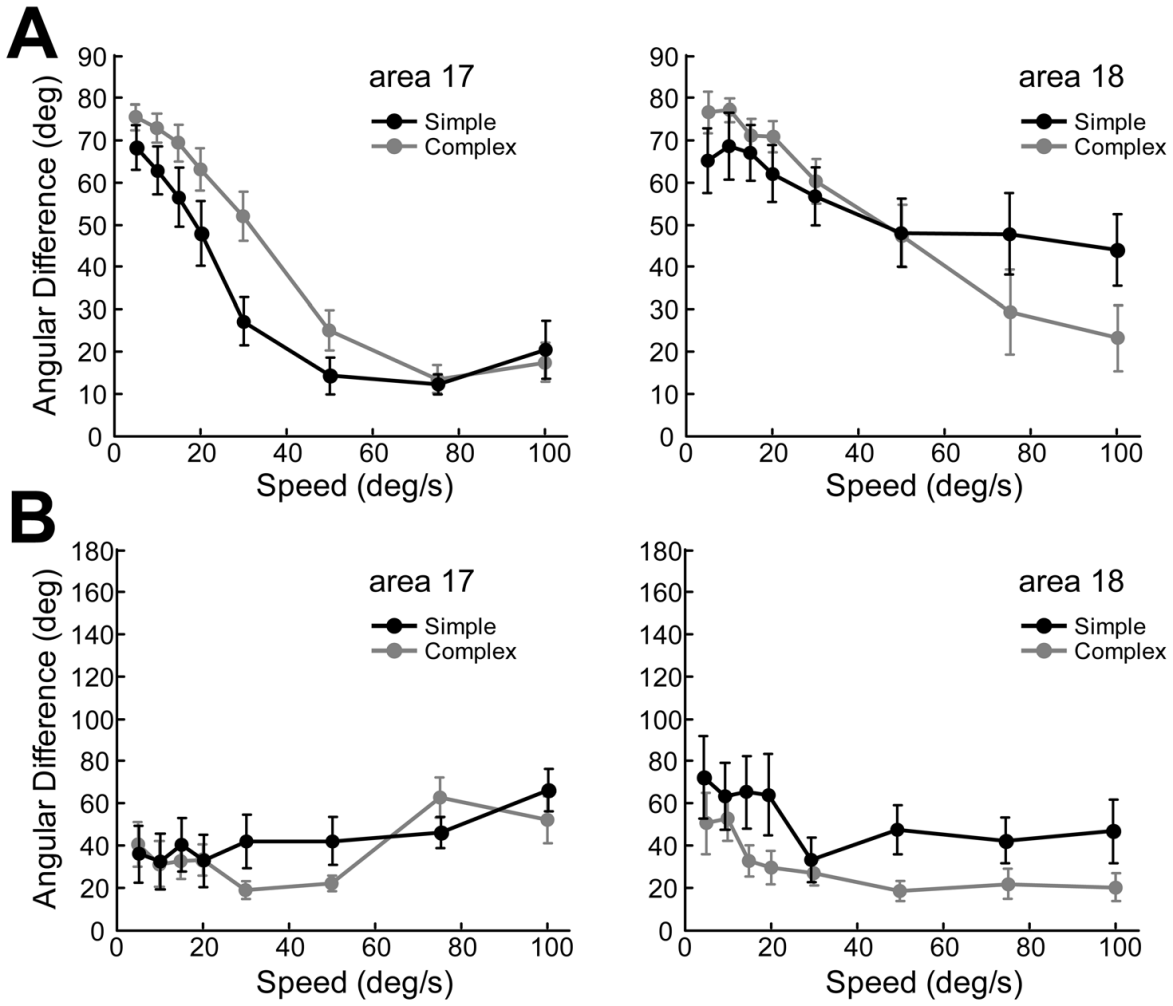
Response properties of simple and complex cells were similar to random-dot motion with different speeds. (A) Angular differences between the preferred motion axes for moving random-dot field and the preferred orientations for sine-wave gratings. (B) Angular differences between the preferred directions for moving random-dot field and for moving sine-wave gratings. Results were calculated across all speeds tested for simple and complex cells in areas 17 and 18 respectively. Error bars represent SEM.

Neurons in cat early visual cortices were conventionally classified into simple and complex cells [61]–[62], and complex cells with nonlinear properties were recently found to integrate visual information with a linear receptive field [63]–[64]. The behavior of simple or complex cells was also found to depend on the actual statistical properties of the visual input [65]. To test whether simple and complex cells are different in the encoding of motion axis information at high speed, response characteristics of simple cells (16 in area 17, 13 in area 18) were compared with those of complex cells (30 in area 17, 21 in area 18). We found that the angular differences for motion axes were not significantly different between simple and complex cells in both areas at all speeds tested (p>0.05, two-way ANOVA, post hoc Bonferroni test) (Figure 14A). Furthermore, the angular differences for directions were also not significantly different between simple and complex cells in both areas at all speeds tested (p>0.05, two-way ANOVA, post hoc Bonferroni test) (Figure 14B). These similarities in the processing of motion axis and direction, as revealed here across different early visual areas, support that the mechanisms underlying random-dot motion processing between simple and complex cells are not fundamentally different [66]–[70]. These results also imply that responses of neurons to random-dot motion in early visual cortices reflect fundamental aspects of early motion processing in addition to those revealed by drifting sine-wave gratings that contain specific orientation information.

### Energy model predicts the motion-axis responses at high speed in both areas

**Figure 15:**
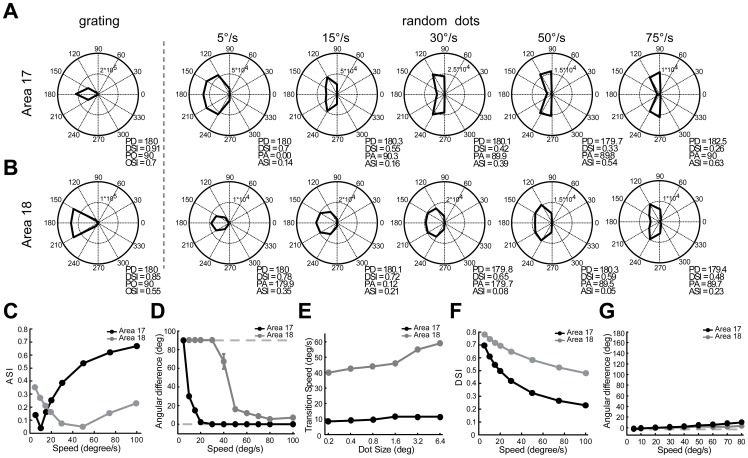
Model simulations for direction-selective cells at both single-cell and population levels. (A–B) Polar plots of direction tunings of direction-selective model cells in areas 17 (A) and 18 (B) to drifting sine-wave gratings and random dots. PD, preferred direction; DSI, direction-selective index; PO, preferred orientation; OSI, orientation-selective index. PA, preferred motion axis; ASI, motion-axis selective index. (C) Simulated motion-axis selective indexes of direction-selective neuronal populations. (D) Angular differences between the fitted orientations of simulated differential population responses to motion axis and the motion axes of the moving-dot stimuli used. The gray broken lines correspond to 0° and 90° angular differences. (E) The influence of dot size on the simulated transition speed. When the dot size increased from 0.2 degree to 6.4 degree, the transition speeds increased from 8.5°/s to 11.5°/s in area 17, and 40°/s to 59°/s in area 18. (F) Simulated direction-selective indexes of direction-selective neuronal populations. (G) Angular differences between the fitted directions of simulated differential population responses to motion direction and the directions of the random-dot stimuli used. The gray broken line corresponds to 0° angular difference. Error bars represent SEM. All population results were averaged from 32 simulated trials, but some error bars were too small to be shown.

Spatio-temporal energy models have been successfully used to explain orientation responses at the population level to moving full-field random-bar stimuli in ferret area 17 [12]–[14], [71] and to predict the motion-axis responses to noise-texture stimuli in macaque V1 and V2 [15]. Therefore, we tested whether the energy model can simulate the responses from direction-selective neurons at both population and single-cell levels in areas 17 and 18. Neuronal responses to different directions and speeds of random-dot stimuli were systematically simulated with RF properties derived from previous and current studies (Table 1). Direction-selective neurons were modeled to prefer only a single direction (Figure 15A–B). Consistent with experimental observations (Figure 11), the preferred motion axes of the simulated neurons changed by 90° between low and high speed (PAs in Figure 15A–B). Furthermore, the preferred directions (calculated with vector summation) of model neurons for random-dot stimuli were the same as those for gratings and were independent of speed (PDs in Figure 15A–B). The motion-axis selective indexes simulated for neuronal populations gradually decreased towards transition speeds and then increased in both areas (Figure 15C). The transition speeds were closely predicted: preferred axis changed 45° at about 10°/s in area 17, but 40°/s in area 18 (Figure 15D). We also simulated the effect of dot size on the transition speeds (Figure 15E). The transition speed changed little in area 17, while showed a considerable increment in area 18 with the increase of dot size, compatible with our recent study in V1 and V2 of monkeys (Figure 10C in An *et al*., 2012). The simulated DSIs in both areas decreased as speed increased (Figure 15F). The DSIs matched the results from electrophysiological recordings in area 17, but were obviously much higher than those in area 18 (comparing Figures 15F and 12D). This discrepancy suggests that direction-selective neurons in area 18 exhibit non-linear response properties to moving dots, while the energy model can only reveal the linear component of the motion responses in area 18. Finally, the model predicted that the preferences of differential population responses to motion direction were independent of speed (Figure 15G), confirming findings revealed by optical imaging (Figures 6 and 10B).

### Summary of early motion processing in areas 17 and 18

**Figure 16:**
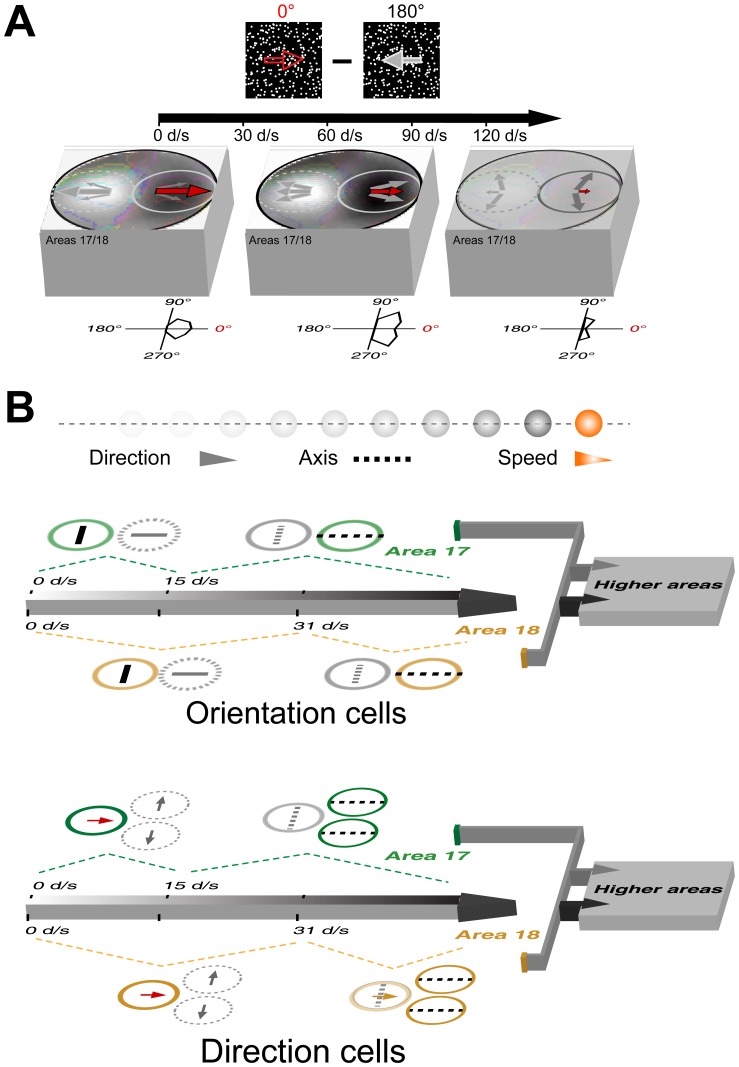
A neuronal mechanism for the processing of random-dot motion in areas 17 and 18. (A) The illustration of how single-neuron behavior accounts for differential population responses. Diagrams of the stimuli (0°–180°) used for generating differential direction responses are shown at the top. The patterns of the differential response images are similar across different speeds, but not the differential response strengths. The solid circles indicate regions that prefer rightward motion (0°), while areas in dotted circles prefer leftward motion (180°). The length of the arrows in the solid/dotted circles represents the response strength of the underlying direction-selective neurons to different directions. The corresponding direction tuning curves under different speeds for neurons preferring a 0° direction are shown as an example at the bottom. (B) Summary of the behavior of orientation- and direction-selective neurons with orthogonal preferences in the processing of the rightward motion at different speeds. The colored circles represent for orientation- and direction-selective neurons with vigorous responses, while those gray circles indicate less active or silent neurons in the processing of the rightward motion below and above the transition speeds. Markers inside these circles indicate stimulus features encoded by the neurons: bar for orientation, dotted line for motion axis, and arrow for direction. Note that direction-selective neurons in area 18 maintain some responses to rightward direction at high speed.

The speed-independent differential direction population responses in fact can be well explained by the behavior of direction-selective neurons. Taking opposite directions of 0° and 180° as an example (Figure 16A), when speed is low, all the direction-selective neurons with a preferred direction of 0° (or 180°) will respond vigorously to random dots moving in the rightward (or leftward) direction, i.e., perpendicular to the preferred orientation of these neurons. At intermediate speeds, the responses of these neurons exhibit two peaks flanking the preferred direction and still contributing to the differential population responses. However, at very high speeds, these neurons will greatly reduce their responses or stop firing to the rightward (or leftward) motion of random dots. Instead, other neurons with direction preferences perpendicular to 0° (or 180°) direction are now activated strongly, thereby encoding the motion-axis information. In other words, direction-selective neurons that prefer upward and downward directions will not respond to rightward and leftward motion at low speed, but do so at high speed. The vigorous responses from these newly activated neurons are cancelled by the subtraction method as the motion-axis information for 0° and 180° directions are the same, resulting in a plain differential direction map (Figure 16A).

In summary, both direction- and orientation-selective neurons in areas 17 and 18 are capable of linearly processing motion information with their response preferences critically depending on motion speed (Figure 16B). Specifically, for rightward motion as an example, the orientation-selective neurons encode the motion axis perpendicular to their preferred orientation at low speed. However, at high speeds, other groups of orientation-selective neurons emerge to encode exclusively the motion axis that is parallel to their preferred orientation. More strikingly, different subsets of direction-selective neurons with orthogonal direction preferences are sequentially activated from low to high speeds. Because areas 17 and 18 have different types of sub-cortical inputs and spatio-temporal frequency preferences, only direction-selective neurons in area 18 will still maintain some responses to their preferred directions at a speed as high as 100°/s, whereas those in area 17 all devote to encode motion axis. Like macaque [72]–[74], the cat visual system is hierarchically organized into multiple functional areas [75]. The outputs of motion information from areas 17 and 18 will project to higher visual areas such as PMLS and 21a for further analysis and integration (Figure 16B), eventually leading to the recognition of object motion before the animal can take appropriate action.

## Discussion

### The basis of early visual motion processing

An abiding challenge in early visual motion processing is how the direction of moving objects within the visual field is determined [2], [76], given that most neurons in early visual cortices are highly orientation and direction selective with small spatio-temporal RFs and precise retinotopic coordinates [37], [77]–[80]. In macaque V1, V2, and V4, orientation-selective neural populations have been recently demonstrated to linearly process motion-axis information by intrinsic-signal optical imaging [15], although this imaging method has limited temporal resolution at second level. When directly exploring population responses at the single-cell level, an early electrophysiological study in cat area 17 has revealed that neuronal populations represent stimulus orientations first perpendicular but later parallel to the motion axis consecutively over a short time window less than 200 ms [10]. All these population studies of different neural signals indicate that orientation-selective mechanism contributes to motion streak processing as proposed by Geisler in human psychophysics [7]. In this study, we found that in addition to orientation-selective neurons, nearly all direction-selective neurons in areas 17 and 18 devoted to encode motion-axis information at high speed. Specifically, direction-selective neurons encoded their preferred motion directions primarily at low speed, but gradually turned to encode motion axes perpendicular to their preferred directions when speed increased. Explicitly, direction-selective neurons in both areas signaled motion direction below a transition speed of 15°/s, but motion axis instead above a transition speed of 31°/s, although direction-selective neurons in area 18 maintained some responses to their preferred directions at this and higher speeds. At intermediate range between 15°/s and 31°/s, the extraction of motion information was achieved by the combination of direction signals processed in area 18 and motion-axis signals encoded in area 17, prior to further analysis in higher visual areas (Figure 16B). This early motion processing strategy reflects the linear filtering property of direction-selective neurons, which have spatio-temporal oriented RFs in areas 17 and 18 for the temporal integration of the series of retinotopic activations induced by moving objects.

### Linear motion processing mechanism in early visual cortices

The neural processing of motion has been extensively studied focusing on the cellular and circuit mechanisms underlying the non-linear generation of direction selectivity of neurons, particularly in primate V1 and MT [2], [73], [81]–[82]. Most previous electrophysiological studies in both cat and primate employed relatively low speeds or temporal frequencies to study the detailed responses or tuning features of direction-selective neurons (See Bradley and Goyal, 2008 for a review). This is mainly because direction-selective neurons tend to exhibit a much weaker, if any, response to their preferred directions when stimuli move at high speed. In earlier studies of simple and complex cells in cat areas 17 and 18, vigorous responses were observed from both types of neurons to motion directions separated apart from the cells' preferred directions when speed gradually increased [66]–[70], [83]–[87]. At high speeds, the two newly evolved response peaks were separated further apart by as much as 90° from the preferred direction. This bimodal response in cat areas 17 and 18 has been nicknamed “the rabbit-ear effect”. Recent studies from cat, ferret, and macaque V1 have demonstrated that the two separate response peaks at high speed actually represent the processing of motion-axis information perpendicular to the neuron's preferred direction [8], [10], [12], [15], [86]. Here in this study at both population and single-cell levels, we have revealed specifically that motion direction is encoded primarily at low speed by direction-selective neurons, whereas above transition speeds, motion axis is encoded instead as the dominant signal by other groups of neurons with orthogonal direction preferences (Figure 16B). Together, in addition to orientation-selective mechanism [7]–[9], [88], our results suggest that direction-selective neurons in different early visual cortices directly contribute to human psychophysical observations of the motion streak or speed line phenomenon. This direction-selective neuronal mechanism associated with speed may serve as a common principle underlying early visual motion processing. As neurons in cat PMLS have large receptive fields and coupled spatial and temporal frequency selectivity [89], PMLS is often considered as analogous to area MT in primates [90]–[92]. Thus, the output motion signals from cat early visual cortices may be selectively combined and further refined in PMLS through a cascade of linear and nonlinear integrative mechanisms.

### Similarities for simple and complex cells and areas 17 and 18 in dot-motion processing

Based on the neural response properties derived from single-unit recording and optical imaging, a spatio-temporal filter model simulated most of the recorded responses, which broadens the usage of the model in predicting responses from direction-selective neurons. The linear filtering property coincides well with previous electrophysiological studies of primary visual cortex on motion processing in both cats and monkeys [8], [10], [59]–[60], [70]. In fact, previous works have suggested that direction selectivity can be understood from the linear filtering property of the spatio-temporal RFs of direction-selective neurons in cat area 17 or 17/18 border and macaque V1 [70], [93]. Many motion models also regard V1 as a “spatio-temporal energy detector” in early motion processing (See Bradley and Goyal, 2008 for a review). Furthermore, previous studies indicate that population activity in ferret area 17 is better described as a single spatio-temporal energy map rather than intersected maps for different visual features [12], [71]. The results on orientation responses of neuronal populations in ferret were later successfully simulated using spatio-temporal energy based models [13]–[14].

Although complex cells are easier to drive by texture stimuli than simple cells, we found no differences between simple and complex cells in both areas 17 and 18 for encoding motion-axis and direction information associated with speed (Figure 14). This is also true regardless of whether the cell is direction or orientation selective. Our results suggest that the mechanisms of motion processing are not fundamentally different between simple and complex cells when studied using moving random-dot stimuli, supporting the absence of discrete simple and complex cell classes in early visual cortex [94]–[97]. Unlike primate V2 which is serially and hierarchically projected to by V1 [73], [98], cat area 17 receives both X- and Y-cell, but predominantly X-cell inputs from the dorsal lateral geniculate nucleus (dLGN); whereas area 18 receives most of its input from Y cells in the dLGN [99]–[102] and thus is generally regarded as a primary motion processing area. This difference in input is the main reason for neurons in area 18 preferring lower SFs and higher TFs than neurons in area 17 [26], [44], [52]. The transition speed in area 18 is therefore much higher than that in area 17 as revealed at both population and single-cell levels. More interestingly, the principle of random-dot motion processing, namely encoding motion direction at low speed, but motion axis at high speed, is generally the same for both areas. This principle, applied to both orientation- and direction-selective neurons across early visual cortices, may well serve the rapid change of motion in the natural world. How this early motion processing mechanism contributes to the perception of complex motion in natural scenes awaits future study, as the statistics of natural images are much more complicated than those of stimuli used in laboratories [103]–[105].

### Concluding remarks

Recognizing a moving object must engage interactions between multiple brain systems involving the integration of form and motion [106]–[107]. Early visual cortices such as areas 17 and 18, serve as a gate for information flow to higher-level processing stages. Our results demonstrate that the basic function of motion-detecting neurons at these early levels is to integrate the series of retinotopic activations generated in space and time to enable motion perception. The processing of motion direction primarily at low speed but motion axis above a certain transition speed applies to all types of cells we recorded. This common principle of early motion processing could provide an efficient way to cope with different kinds of motion generated by objects at various speeds. It may also directly contribute to the invariant motion perception of different moving objects and carriers in nature.

## Supporting Information

Figure S1 **The analysis of residual luminance of dots on the monitor.** (A) An image of static dot-grid stimuli. The luminance of the dots was 0.28 cd/m^2^. Note that as the exposure time of the SLR camera was very short, only one line of illuminated dots of the dots grid can be clearly seen. The intensity profile of pixels in a line crosses the center of the dots as pointed by the white arrows was shown below. (B) An image of rightwards moving dot-grid stimuli with a speed of 200°/s. We used dot-grid stimuli here to better identify the positional change of the dots between frames. Intensity profiles of pixels in the two lines cross the center of dots were shown below. The total intensity of each dot as pointed by white arrows in (A) and (B) was quantified by integrating the intensity in a square window (white square in A and B). The total intensity of each dot in the second line of (B) was significantly lower than that of each dot in (A) (p<0.001, t-test), indicating that the luminance of the dots decreased more than 200 folds in 10 ms (from ∼87 cd/m^2^ to less than 0.28 cd/m^2^).
(TIF)

Figure S2 **Motion-axis responses to moving random-dot stimuli with different background luminance.** (A) Image of the cortical vasculature. The irregular green polygon defines the selected region of area 17 for further analysis. A, Anterior; L, lateral. Scale bar: 1 mm. (B) Differential orientation map (45°–135°) and the corresponding result of response profile analysis acquired using sine-wave grating stimuli. (C–D) Differential motion-axis maps (135°–45°) and the corresponding results of response profile analysis obtained by using random-dot stimuli with background luminance of 15 cd/m^2^ and 0.2 cd/m^2^, respectively. Under the same speed, the response profiles were almost identical in shape between the two background conditions. Colored iso-orientation contours were derived from the orientation preference map for sine-wave grating stimuli and were superimposed on the gray images. The red curves in the plots represent the best fitting cosine functions. Scale bar: 1 mm.
(TIF)

Figure S3 **Single-unit responses in area 17 to moving random-dot stimuli with different background luminance.** (A) Polar plot of direction tuning of a direction-selective cell generated using sine-wave grating stimuli. N = 5 trials. (B) Polar plots of direction tunings acquired by using random-dot stimuli with background luminance of 0.2 and 15 cd/m^2^, respectively. N = 10 trials. (C) Angular differences between the preferred orientations for sine-wave gratings and the preferred motion axes for moving random dots. The angular difference was significantly changed only with the speed (p<0.01, two-way ANOVA; n = 14 cells), but not with the luminance of the background (p = 0.73, two-way ANOVA). Error bars represent SEM.
(TIF)
