# Supplementary figures and images for: The Mechanism for Processing Random-Dot Motion at Various Speeds in Early Visual Cortices
Source: PLoS One. 2014 Mar 28;9(3):e93115. doi: 10.1371/journal.pone.0093115 (PMC3969330; doi:10.1371/journal.pone.0093115)

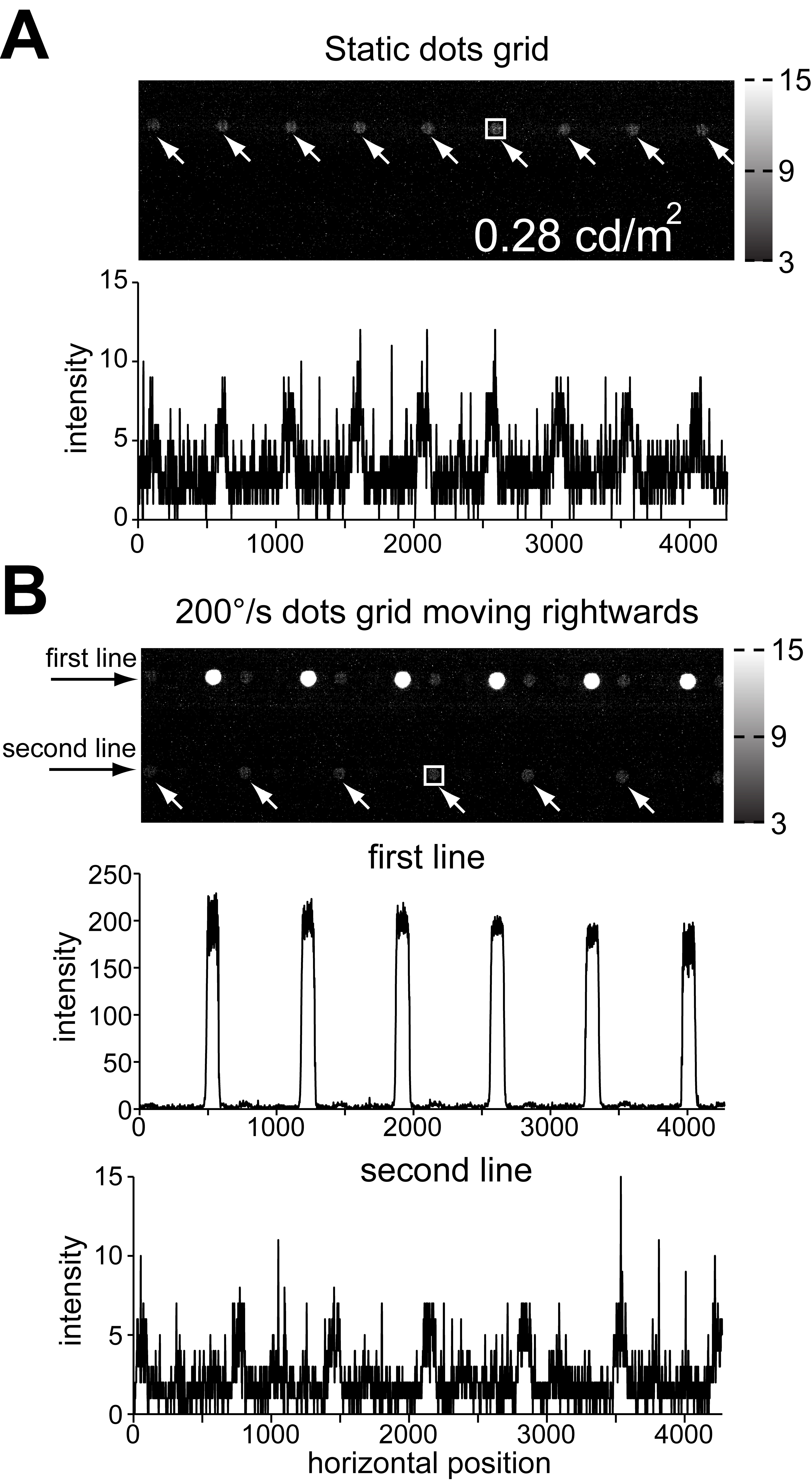

Supplement: Figure S1 — The analysis of residual luminance of dots on the monitor. (A) An image of static dot-grid stimuli. The luminance of the dots was 0.28 cd/m2. Note that as the exposure time of the SLR camera was very short, only one line of illuminated dots of the dots grid can be clearly seen. The intensity profile of pixels in a line crosses the center of the dots as pointed by the white arrows was shown below. (B) An image of rightwards moving dot-grid stimuli with a speed of 200°/s. We used dot-grid stimuli here to better identify the positional change of the dots between frames. Intensity profiles of pixels in the two lines cross the center of dots were shown below. The total intensity of each dot as pointed by white arrows in (A) and (B) was quantified by integrating the intensity in a square window (white square in A and B). The total intensity of each dot in the second line of (B) was significantly lower than that of each dot in (A) (p<0.001, t-test), indicating that the luminance of the dots decreased more than 200 folds in 10 ms (from ∼87 cd/m2 to less than 0.28 cd/m2). (TIF) [file pone.0093115.s001.tif]

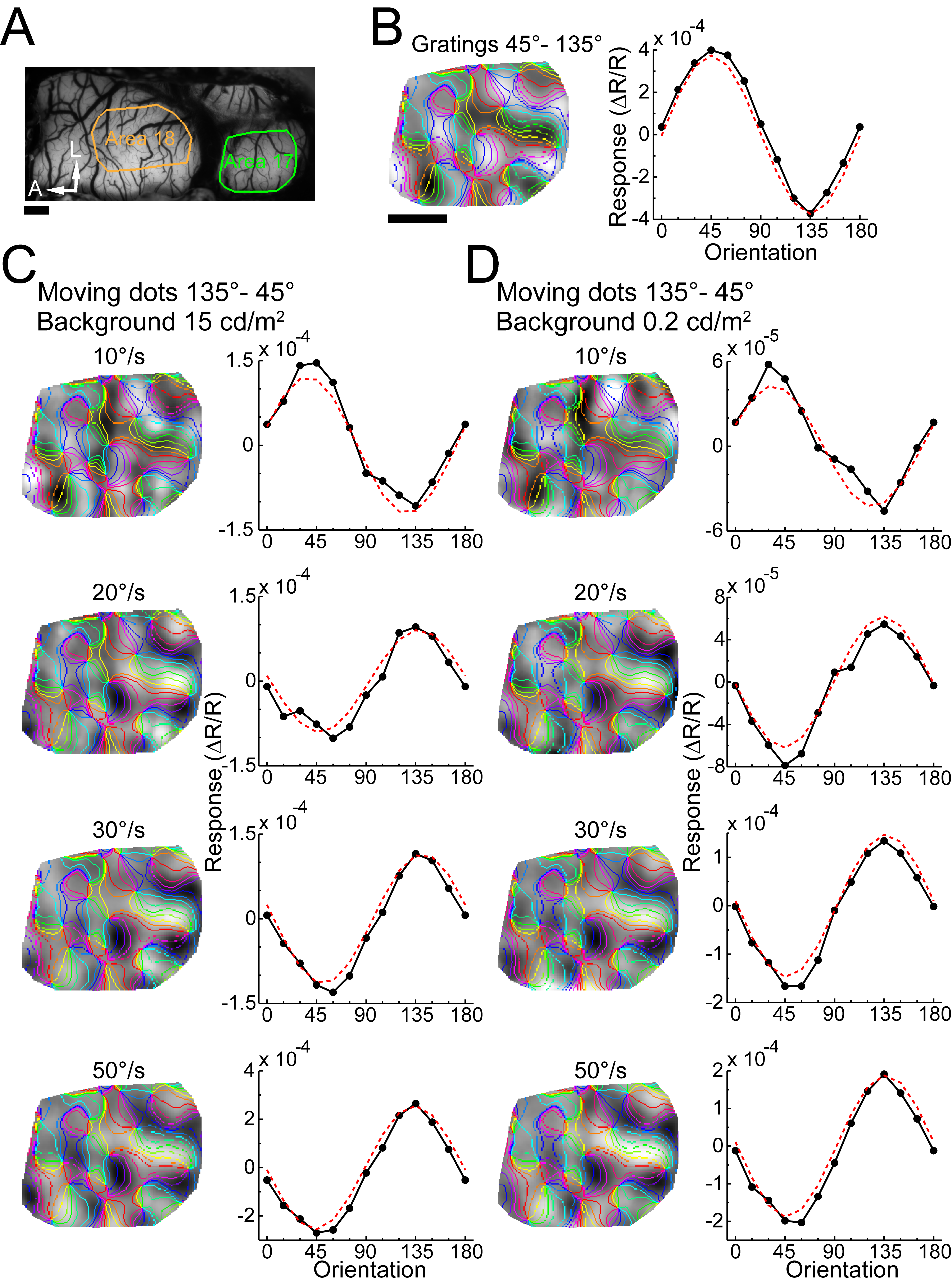

Supplement: Figure S2 — Motion-axis responses to moving random-dot stimuli with different background luminance. (A) Image of the cortical vasculature. The irregular green polygon defines the selected region of area 17 for further analysis. A, Anterior; L, lateral. Scale bar: 1 mm. (B) Differential orientation map (45°–135°) and the corresponding result of response profile analysis acquired using sine-wave grating stimuli. (C–D) Differential motion-axis maps (135°–45°) and the corresponding results of response profile analysis obtained by using random-dot stimuli with background luminance of 15 cd/m2 and 0.2 cd/m2, respectively. Under the same speed, the response profiles were almost identical in shape between the two background conditions. Colored iso-orientation contours were derived from the orientation preference map for sine-wave grating stimuli and were superimposed on the gray images. The red curves in the plots represent the best fitting cosine functions. Scale bar: 1 mm. (TIF) [file pone.0093115.s002.tif]

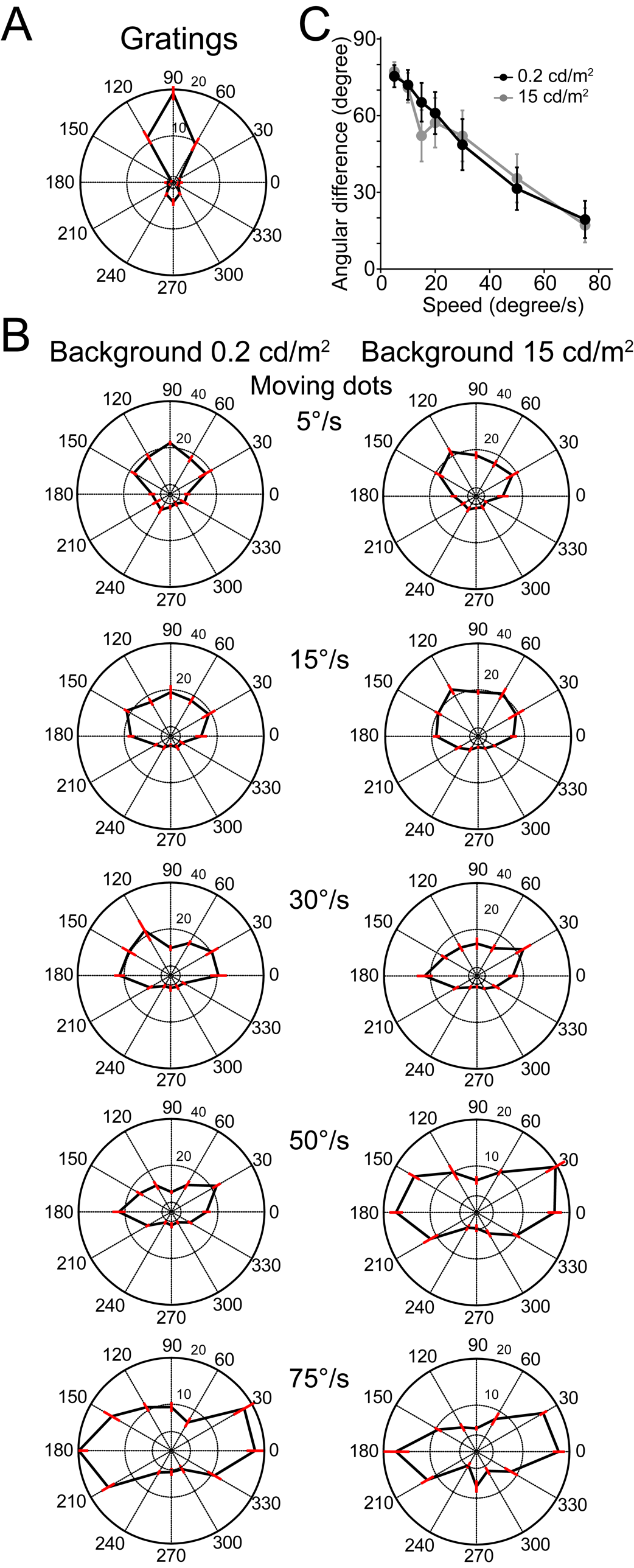

Supplement: Figure S3 — Single-unit responses in area 17 to moving random-dot stimuli with different background luminance. (A) Polar plot of direction tuning of a direction-selective cell generated using sine-wave grating stimuli. N = 5 trials. (B) Polar plots of direction tunings acquired by using random-dot stimuli with background luminance of 0.2 and 15 cd/m2, respectively. N = 10 trials. (C) Angular differences between the preferred orientations for sine-wave gratings and the preferred motion axes for moving random dots. The angular difference was significantly changed only with the speed (p<0.01, two-way ANOVA; n = 14 cells), but not with the luminance of the background (p = 0.73, two-way ANOVA). Error bars represent SEM. (TIF) [file pone.0093115.s003.tif]
